# Stromal BMP signalling maintains stem cell genome integrity and prevents gastric cancer initiation

**DOI:** 10.1038/s41419-026-09088-z

**Published:** 2026-07-17

**Authors:** Zihao Li, Zhengting Wang, Ruichen Yang, Jiarui Zhao, Shaoyang Zhang, Liuchun Feng, Haiyu Shen, Hua Yue, Zhenlin Zhang, Xiaolin Guo, Dengli Hong, Yuji Mishina, Ye-Guang Chen, Qiang Wu, Huijuan Liu, Baojie Li

**Affiliations:** 1 Bio-X Institutes, Shanghai Jiao Tong University, Shanghai, China; 2 Department of Gastroenterology, Ruijin Hospital, School of Medicine, Shanghai Jiao Tong University, Shanghai, China; 3 Department of Osteoporosis and Bone Diseases, Shanghai Clinical Research Center of Bone Diseases, Shanghai Jiao Tong University of Medicine Affiliated Sixth People’s Hospital, Shanghai, China; 4 Institute for Translational Medicine on Cell Fate and Disease, Shanghai Ninth People’s Hospital, Key Laboratory of Cell Differentiation and Apoptosis of Ministry of Education, Department of Pathophysiology, Shanghai Institute of Hematology, Ruijin Hospital, Shanghai Jiao Tong University School of Medicine, Shanghai, China; 5 School of Dentistry, University of Michigan, Ann Arbor, MI USA; 6 State Key Laboratory of Membrane Biology, Tsinghua-Peking Center for Life Sciences, School of Life Sciences, Tsinghua University, Beijing, China; 7 The State Key Laboratory of Mechanism and Quality of Chinese Medicine, Macau University of Science and Technology. Avenida Wai Long, Taipa, Macau SAR China; 8 Shanghai Institute of Stem Cell Research and Clinical Translation, Shanghai, China

**Keywords:** Gastric cancer, Cancer stem cells, Cancer microenvironment

## Abstract

Gastric cancer primarily originates from gastric stem/progenitor cells and is driven by somatic mutations. Although *Helicobacter pylori* is the main risk factor, how it drives malignancy is still not well understood. Analysis of single-cell RNA sequencing data reveals that human gastric cancer correlates with suppressed BMP signalling, a crucial niche signal for gastric stem cells, in stromal cells rather than epithelial cells. Genetic disruption of BMP signalling in *Col1a2*^*+*^ stromal cells or *Acta2*^*+*^ myocytes/pericytes alone, but not in gastric stem cells themselves, triggers mutations in gastric stem cells and initiates cancer development. Mechanistically, loss of BMP signalling increases stromal production of Wnt ligands, which dose-dependently drive transcription-replication collisions, R-loops, and DNA damage in gastric stem cells. The resulting DNA damage and carcinogenesis can be prevented by small-molecule inhibitors targeting Wnt pathway. Importantly, we identify inflammation as a key disruptor of stromal BMP signalling, as seen in patient samples with chronic atrophic gastritis and *H. pylori*-infected mouse gastric samples, which is associated with DNA damage. Together, these findings show how chronic inflammation derails niche signalling to drive stem cell mutations and gastric cancer and highlight promising avenues for early prevention.

## Introduction

Gastric cancer is a major global cause of cancer and death, driven by somatic mutations [1]. These mutations can result from intrinsic factors or extrinsic exposures such as radiation, genotoxic agents, and infections [1–4]. About 25% of cancer cases are linked to inflammation, including gastric cancer (GC) [5], one of the most prevalent and deadly cancers [6, 7]. Genomic sequencing has revealed that most GC cases harbour mutations [8–10], and display diverse mutational signatures [11–13]. The primary risk factor for GC is *Helicobacter pylori* (*H. pylori*) infection, which promotes cancer development through a progression from non-atrophic gastritis (NAG) to chronic atrophic gastritis (CAG), intestinal metaplasia (IM), and dysplasia [12, 14]. It is generally believed that *H. pylori* virulence factors cause GC by activating pro-proliferative signalling pathways, causing DNA damage, and triggering inflammation [15–18], and inflammation further drives GC by promoting cell proliferation through cytokine production and inducing DNA mutations via reactive oxygen species (ROS) [19]. However, the long latency-often decades-between *H. pylori* infection and GC onset, the frequent absence of *H. pylori* in GC and precancerous tissues, and the occurrence of GC even after *H. pylori* eradication [20, 21], indicate that additional, as-yet-unidentified mechanisms contribute to GC development [18, 22].

GCs are believed to originate primarily from gastric stem/progenitor cells (GSCs) located in the lower part of the gastric glands [23–26]. GSC proliferation and differentiation are regulated by their niche, particularly stromal cells. These stromal cells secrete pro-proliferative Wnt and R-spondin (Rspo) signals, establishing a gradient from the gland base toward the lumen [27, 28], and they also produce pro-differentiation bone morphogenetic proteins (BMPs), which form an opposing gradient [29–32]. BMPs bind to type II receptors, which phosphorylate and activate type I receptors (BMPRIA and BMPRIB). The activated type I receptors then phosphorylate Smad1/5, allowing them to form complexes with Smad4. These complexes translocate to the nucleus and regulate the expression of target genes, including *ID1*, *ID2*, *ID3*, and *ID4* [33]. BMP signalling has been shown to exhibit tumour-suppressive activity in several epithelial cancers by promoting differentiation, limiting proliferation, and dampening inflammation [28, 32, 33]. Disruption of BMP signalling in *Axin2*^*+*^ or *Lgr5*^*+*^ GSCs leads to gastric hyperplasia that does not progress to dysplasia or neoplasia [34, 35]. Notably, disruption of BMP signalling in stromal cells from early embryonic stages using *Foxl1-Cre* or *Dmp1-Cre* mice also results in gastric hyperplasia that does not progress to dysplasia or neoplasia, although the underlying mechanisms remain unclear [36, 37]. Given the rarity of GC in young adults, the marked heterogeneity of stromal cells, and the presence of multiple BMP-responsive cell types, the precise contribution of BMP signalling to GC development remains unclear and warrants further investigation [38].

Our analysis of patient single cell RNA-seq (scRNA-seq) data shows that gastric cancer development correlates with reduced BMP signalling in stromal, but not epithelial, cells. In adult mice, selective disruption of BMP signalling in *Col1a2*^+^ stromal cells or *Acta2*^+^ myocytes/pericytes, but not in *Lgr5*^+^ GSCs, is sufficient to drive invasive gastric cancer, driven by somatic mutations in GSCs. This is a surprising finding, because gastric cancer in mice usually requires combined deletion of multiple genes or treatment with mutagens together with *H. pylori* infection [23, 39]. Loss of stromal BMP signalling increases Wnt/R-spondin production, which dose-dependently drive transcription-replication collisions, R-loops, and DNA damage in GSCs. This challenges the view that mutations are purely random events that simply accumulate with each stem cell division. We further identify inflammation as a critical factor that disrupts stromal BMP signalling and triggers DNA damage, from chronic atrophic gastritis through to gastric cancer progression. Together, these findings reveal a chronic inflammation-driven mutagenic mechanism that initiates GSC transformation and gastric cancer and highlight potential targets for early cancer prevention.

## Results

### Stromal but not epithelial BMP signalling correlates with human GC progression

**Fig. 1:**
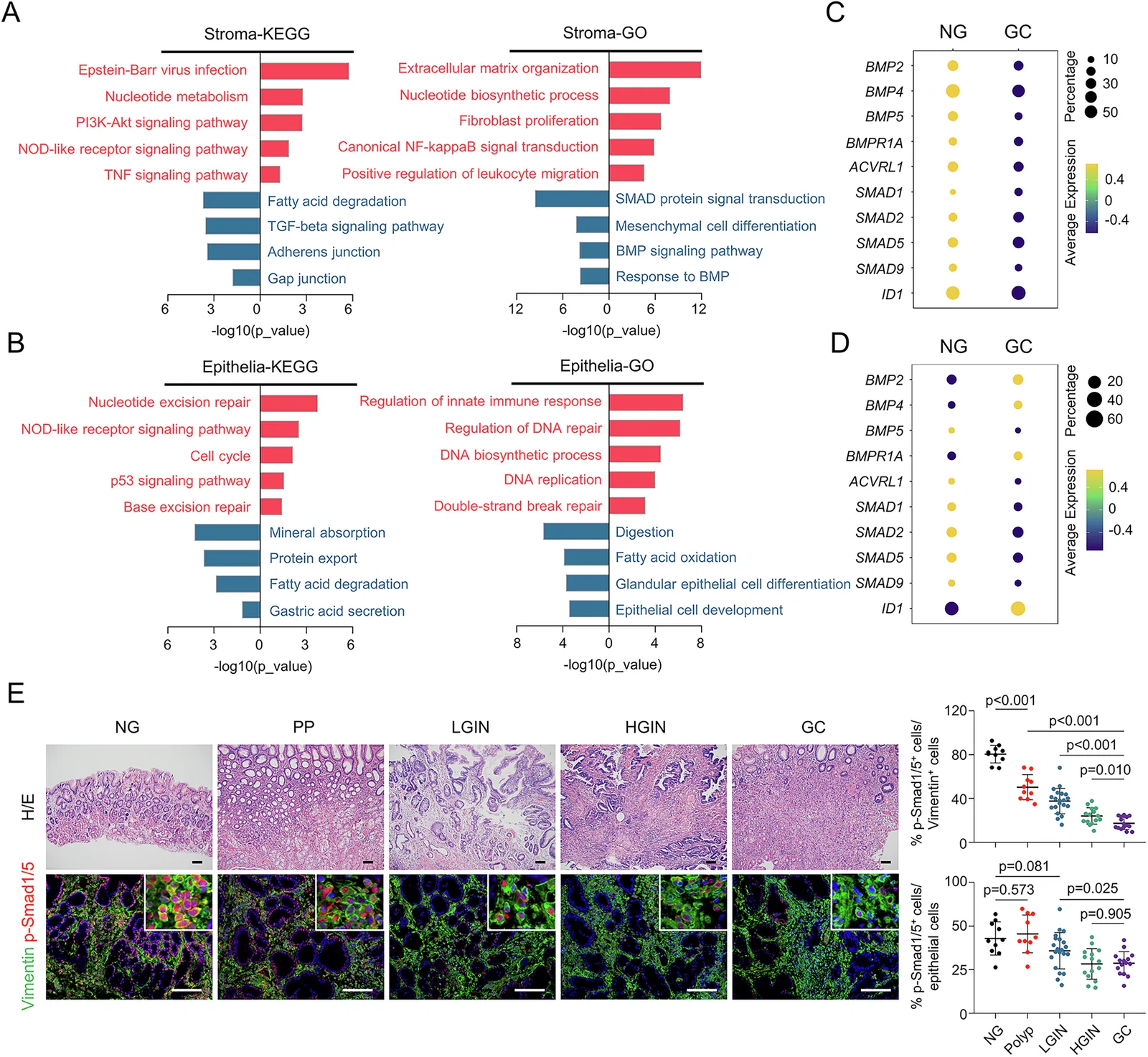
Stromal rather than epithelial BMP signalling is inversely correlated to human GC development and progression. **A** scRNA-seq analysis of pathways and modules altered in the stromal cells of patient gastric cancer samples (GSE183904). NG = 11, GC = 29. **B** scRNA-seq analysis of pathways and modules altered in the epithelial cells of patient gastric cancer samples (GSE183904). NG = 11, GC = 29. **C** The expression levels of major BMP ligands, signalling molecules, and target genes in the stromal cells of patient gastric cancer samples. NG = 11, GC = 29. **D** The expression levels of major BMP ligands, signalling molecules, and target genes in the epithelial cells of patient gastric cancer samples. NG = 11, GC = 29. **E** Co-immunostaining results for p-Smad1/5 and Vimentin on patient sample sections. The enlarged views represent Vimentin/p-Smad1/5 staining result. Right panels: quantification of the data. NG, *n* = 10; PP, *n* = 10; LGIN, *n* = 20; HGIN, *n* = 15; GC, *n* = 15. Unpaired two-tailed Student’s *t* test was applied for (**E**). *p* < 0.05 was considered statistically significant. Scale bars: 100 μm.

Previous mouse model studies have shown that BMPs suppress GSC proliferation directly or through stromal cells. However, the precise role for BMP signalling in human GC development and progression remains underexplored [7, 13]. We analysed single-cell RNA sequencing (scRNA-seq) data from patient GC samples, broadly classifying cells into epithelial, endothelial, stromal, T cell, B cell, mast cell, myeloid, and plasma cell populations (Supplementary Fig. S1A, B). KEGG and GO analyses revealed that stromal cells exhibited increased expression of genes related to TNF signalling, NOD-like receptor signalling, NF-κB signalling, extracellular matrix, and proliferation, and decreased expression of genes related to BMP signalling, TGFβ signalling, differentiation, and adherens junction (Fig. 1A). Epithelial cells showed enriched expression in cell division, inflammation, and DNA damage and repair, and decrease expression of genes related to digestion, differentiation, mineral absorption, and gastric acid secretion (Fig. 1B). Expression of many of the BMP pathway genes was consistently suppressed in stromal cells, but not in epithelial cells of the GC samples (Fig. 1C, D).

To verify this, we collected 60 patient samples, including 10 polyps (PP), 20 low-grade intraepithelial neoplasia (LGIN), 15 high-grade intraepithelial neoplasia (HGIN), and 15 gastric cancer samples (GC), along with 10 normal gastric tissue (NG, para-tumour tissue) samples. Co-immunostaining revealed an activation of BMP/Smad signalling in stromal cells, evidenced by the presence of p-Smad1/5 in Vimentin^+^ stromal cells, which decreased progressively in samples of late stages (Fig. 1E). However, this trend was not observed in epithelial cells (Fig. 1E). These results suggest that BMP signalling may play a more critical role in human GC development and progression in stromal cells rather than in epithelial cells.

### Genetic tracing of several stromal lineages in antrum and corpus

**Fig. 2:**
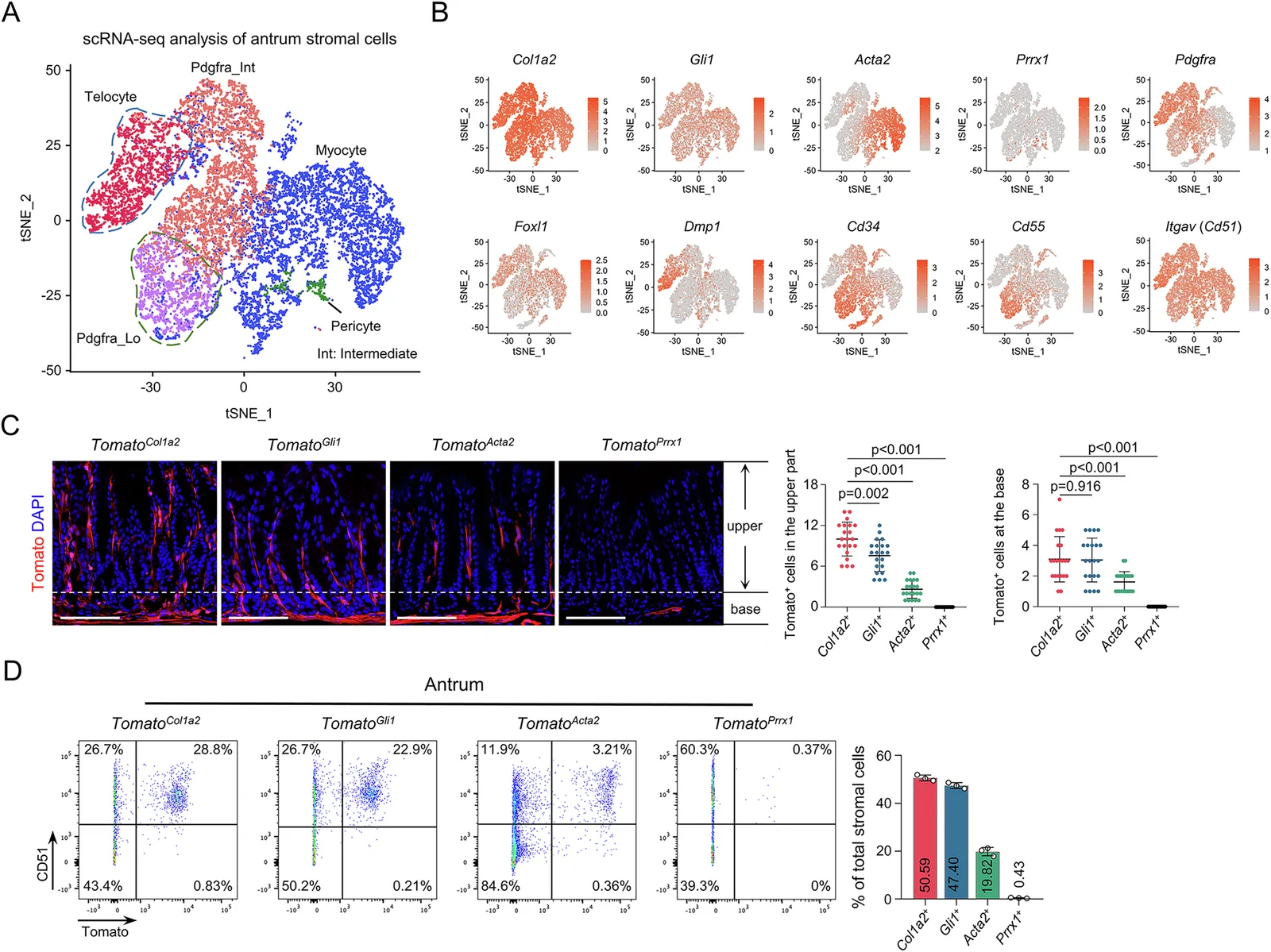
Genetic tracing of several stromal lineages in antrum. **A** scRNA-seq analysis classified stromal cells of mouse antrum (GSE224737) into 5 distinct populations. **B** tSNE analysis of the expression of stromal cells markers including *Itgav* (*CD51*). **C** Representative genetic tracing results for the antrum are shown. The antrum from adult male mice that received TAM one month prior was sectioned and stained with DAPI. Right panels: Quantitative data of Tomato^+^ cells per gland in the upper part and the base of the glands. *n* = 21, with 7 views each of 3 mice. White dashed lines indicate the boundary between the base and the upper region. **D** Quantitation of flow cytometry results showing the proportions of the four stromal lineages (Tomato^+^ cells among total CD31^-^CD45^-^EpCAM^-^CD51^+^ stromal cells). *n* = 3 mice. Unpaired two-tailed Student’s *t* test was applied for (**C**). *p* < 0.05 was considered statistically significant. Scale bars: 100 μm.

To investigate how stromal BMP signalling contributes to GC development, we sought to perturb BMP signalling by ablating the relevant gene(s) specifically in stromal cells. Since stromal cells are heterogeneous [12], we analysed the scRNA-seq data and found that mouse stromal cells in the antrum could be classified into telocyte (*Pdgfra*^*high*^), *Pdgfra*^*int*^, *Pdgfra*^*low*^, myocyte, and pericyte populations (Fig. 2A and Supplementary Fig. S2A), consistent with previous reports [29, 40]. We further show that the expression of stromal markers such as *Col1a2*, *Gli1*, *Acta2*, *Prrx1*, *Pdgfra*, *Foxl1*, *Dmp1, CD34*, *CD55*, and *CD51* was observed across multiple populations rather than being restricted to a specific group (Fig. 2B). Moreover, the expression of *Dmp1* and *Foxl1* was limited, which may lead to underestimation of the effect of stromal BMP signalling [36, 37]. Currently, no specific genetic markers can reliably distinguish these subgroups in vivo. We therefore used *Col1a2*, *Gli1*, *Acta2*, and *Prrx1* to capture overlapping fractions of the stromal compartment, as well as certain more defined subpopulations. *Col1a2* labels the majority of stromal cells across all five populations; *Gli1* has been used to mark stromal niche cells in the gastrointestinal tract; *Acta2* marks pericytes and myocytes; and *Prrx1* labels a subset of pericytes.

Genetic tracing using adult *Tomato*^*Col1a2*^, *Tomato*^*Gli1*^, *Tomato*^*Acta2*^, and *Tomato*^*Prrx1*^ mouse lines confirmed that *Col1a2* marked the largest proportion of stromal cells, followed by *Gli1*, *Acta2*, and *Prrx1*, in both the antrum and corpus (Fig. 2C and Supplementary Fig. S2B). This was further validated through flow cytometry analysis of Tomato^+^ cells within total stromal cells (Fig. 2D and Supplementary Fig. S2C). *Col1a2*, *Gli1*, and *Acta2* were found to label the muscularis mucosae and stromal cells at the base, with notable differences in labelling primarily occurring in the upper part of the antrum (Fig. 2C). Immunostaining demonstrated that Tomato^+^ stromal cells were negative for CD31, CD45, or EpCAM and that αSMA or Gli1 was expressed in subsets of *Col1a2*-lineage stromal cells (Supplementary Fig. S2D, E).

### Stromal *Bmpr1a* ablation led to expansion of gastric stem cells

**Fig. 3:**
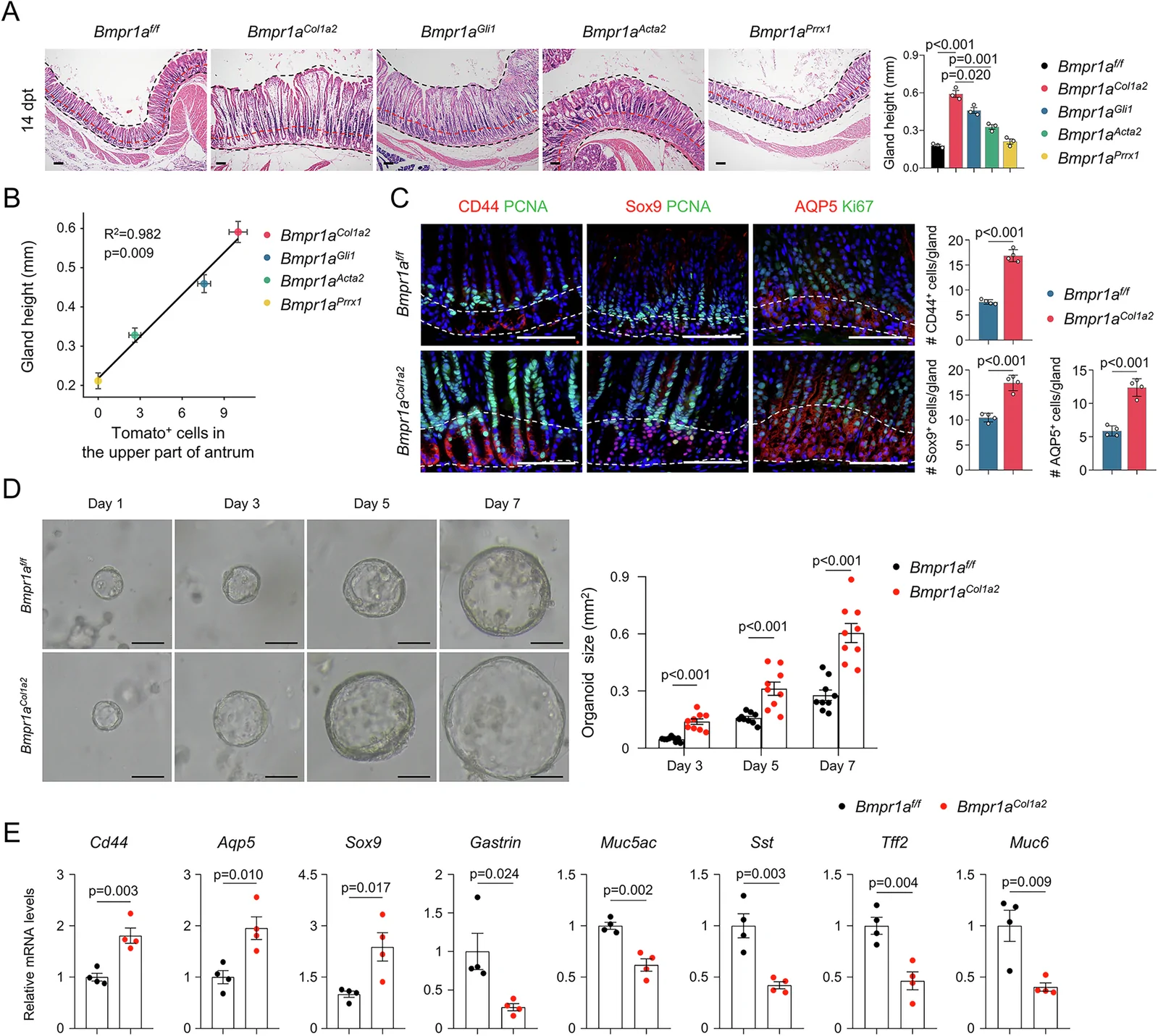
Stromal *Bmpr1a* ablation led to expansion of gastric stem cells. **A** Representative H/E staining results of antrum sections on Day 14 after TAM administration. Right panels: quantitative data of gland height (mm), *n* = 3 mice. Dpt: days post TAM. **B** Correlations between gland height and stromal population size in the upper part of antrum. *n* = 3 mice for each genotype. **C** Immunostaining for GSC and proliferation markers in antrum sections of *Bmpr1a*^*Col1a2*^ and control mice (14 days post TAM). Right panels: quantitation data. *n* = 4 mice. **D** Co-culture experiments showed that *Bmpr1a*^*−/−*^ stromal cells had greater ability in promoting antral organoid growth. Tomato^+^ stromal cells were isolated form *Tomato*^*Col1a2*^ or *Bmpr1a; Tomato*^*Col1a2*^ mice by FACS sorting. Right panels: quantitation results of the organoid size. *n* = 9 (triplicate with 3 repeats). **E** qPCR analysis of the expression of *Cd44*, *Aqp5*, *Sox9*, *Gastrin*, *Muc5ac*, *Sst*, *Tff2* and *Muc6* in organoids co-cultured with wild-type or *Bmpr1a*^*−/−*^ stromal cells. *n* = 4 mice. Unpaired two-tailed Student’s *t* test was applied for (**A**), (**C**), (**D**) and (**E**). Linear regression analysis was applied for (**B**). *p* < 0.05 was considered statistically significant. Scale bars: 100 μm.

To disrupt BMP signalling in stromal cells, we generated *Bmpr1a*^*Col1a2*^, *Bmpr1a*^*Gli1*^, *Bmpr1a*^*Acta2*^, and *Bmpr1a*^*Prrx1*^ mice to induce *Bmpr1a* ablation in distinct subsets of stromal cells in adult mice. We focused on *Bmpr1a* because it encodes BMPR1A, a key mediator of BMP signalling implicated in gastric cancer pathogenesis (Fig. 1C) [41]. Fourteen days after TAM administration, we noted a marked reduction in p-Smad1/5 signals in Vimentin^+^ stromal cells in antrum sections (Supplementary Fig. S3A). We observed increases in both the height of the antrum glands and the number of PCNA^+^ proliferating cells in all 4 mouse lines except *Bmpr1a*^*Prrx1*^ mice by 14 days after TAM administration (Fig. 3A and Supplementary Fig. S3B), indicating a hyperplasia phenotype. Immunostaining for the G-cell marker gastrin and quantitative PCR analysis of epithelial type-specific markers revealed that epithelial differentiation was impaired (Supplementary Fig. S3B, C). Interestingly, in all four mouse lines, antrum height showed a stronger positive correlation with the abundance of stromal lineages in the upper region than in the basal region (Fig. 3B and Supplementary Fig. S3D). While it is established that the stromal niche surrounding GSCs secretes signalling molecules to drive GSC proliferation [27, 29, 40], our findings suggest that stromal lineages in the upper part quantitatively constrain gastric epithelial proliferation in response to local BMPs.

qPCR analysis revealed increased expression of the GSC markers CD44, AQP5, and SOX9 in antral samples from *Bmpr1a*^*Col1a2*^ mice (Supplementary Fig. S3E), which are also commonly used as markers of gastric hyperplasia and cancer [23, 25, 26, 42–44]. Immunostaining confirmed increased numbers of GSCs positive for CD44, Sox9, or AQP5, at the base (Fig. 3C). To further confirm the effect of *Bmpr1a*-ablated stromal cells on GSCs, we co-cultured gastric organoids, a commonly used in vitro model for studying GSCs, with *Bmpr1a*^*-/-*^ or wild-type stromal cells. These assays showed that *Bmpr1a*^*-/-*^ stromal cells more strongly promoted organoid growth, suppressed differentiation, and increased the expression of *Cd44*, *Aqp5*, and *Sox9* compared with wild-type stromal cells (Fig. 3D, E), consistent with our in vivo findings. These results suggest that the ablation of *Bmpr1a* in stromal cells activates GSCs at the antrum base, likely through a paracrine mechanism.

### Stromal *Bmpr1a* ablation led to invasive GC following hyperplasia

**Fig. 4:**
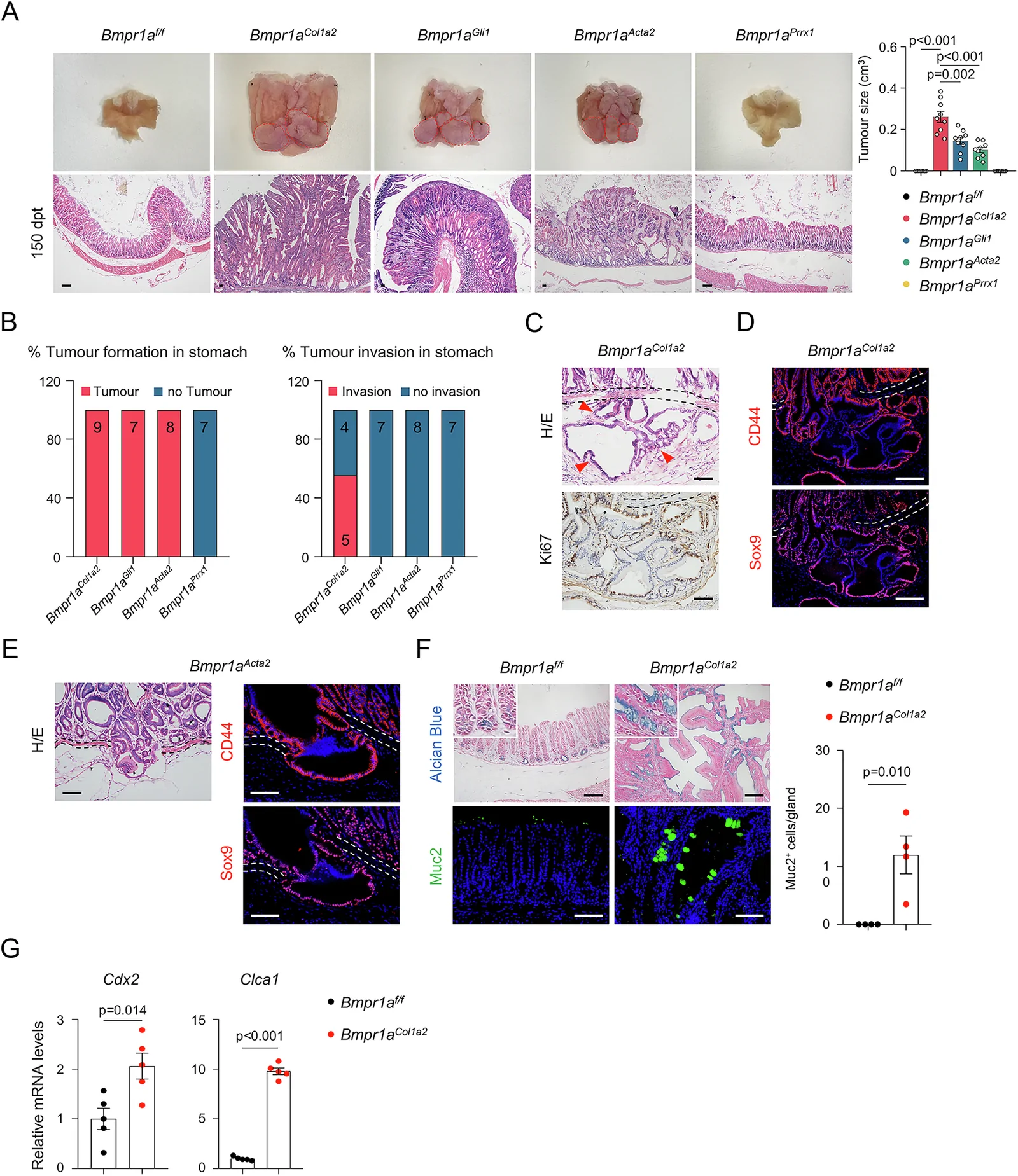
*Bmpr1a* ablation in *Col1a2*^+^ or *Acta2*^+^ cells induces invasive GC. **A** Representative images and H&E staining of spontaneous gastric tumours from the four *Bmpr1a*-ablated mouse lines on day 150 after TAM administration. Right panels: quantification of tumour size (*n* = 9 tumours from 4 mice for each genotype). **B** Percentages of the mice with tumours or invasive tumours 5 months post TAM administration. *Bmpr1a*^*Col1a2*^: *n* = 9, *Bmpr1a*^*Gli1*^: *n* = 7, *Bmpr1a*^*Acta2*^: *n* = 8, *Bmpr1a*^*Prrx1*^: *n* = 7. **C** Representative H/E staining and Ki67 immunostaining results of invasive tumours in *Bmpr1a*^*Col1a2*^ mice. **D** Representative immunostaining results for CD44 or Sox9 in invasive tumours from *Bmpr1a*^*Col1a2*^ mice. **E** Representative H/E staining and immunostaining results for CD44 or Sox9 of invasive tumours in *Bmpr1a*^*Acta2*^ mice 7 months post TAM administration. **F** Representative results of Alcian blue staining and Muc2 immunostaining of invasive tumours from *Bmpr1a*^*Col1a2*^ mice. Right panels: quantitation results of Muc2^+^ cells per gland. *n* = 4 mice. **G** qPCR analysis of the expression of intestine-specific genes in antrum samples from *Bmpr1a*^*Col1a2*^ mice. *n* = 5 mice. Unpaired two-tailed Student’s *t* test was applied for (**A**), (**F**) and (**G**). *p* < 0.05 was considered statistically significant. Scale bars: 100 μm.

Over time, some of the *Bmpr1a*-deficient mouse lines developed tumours. *Bmpr1a*^*Gli1*^ mice survived up to 5 months, while *Bmpr1a*^*Col1a2*^ mice survived up to 9 months. In contrast, *Bmpr1a*^*Acta2*^ and *Bmpr1a*^*Prrx1*^ mice survived beyond 9 months and exhibited no or only mild phenotypes. Accordingly, 9 months was used as the experimental endpoint. Five months after *Bmpr1a* ablation in adult mice, hyperplasia was observed in the antrum of all mouse lines except the *Bmpr1a*^*Prrx1*^ mice (Fig. 4A, B). Polyps were observed in *Bmpr1a*^*Col1a2*^, *Bmpr1a*^*Gli1*^, *Bmpr1a*^*Acta2*^ mouse lines even 3 months after induced *Bmpr1a* ablation (Supplementary Fig. S3F). These phenotypes are more severe than those in the *Dmp1*-Cre; *Bmpr1a*^*f/f*^ mice and the *Foxl1*-Cre; *Bmpr1a*^*f/f*^ mice at 3–5 months of age [36, 37], likely due to the difference in the abundance of these cell lineages.

Notably, 56% of *Bmpr1a*^*Col1a2*^ mice (*n* = 9) developed tumours that penetrated the muscularis mucosa and invaded the submucosa by 5 months post TAM administration, increasing to 100% by 9 months (*n* = 7) (Fig. 4B, C). Each affected mouse exhibited 2-3 malignant tumours. The invasive tumours expressed CD44 and Sox9 (Fig. 4D). Additionally, 2 of 3 *Bmpr1a*^*Acta2*^ mice developed invasive gastric tumours by 7 months after TAM administration, suggesting that GC development is slower when *Bmpr1a* is ablated in a low-abundance stromal lineage (Fig. 4E). Alcian blue staining revealed the presence of intestine-specific goblet cells within these tumours, which was further confirmed by immunostaining for Muc2, an intestinal marker, and qPCR analysis of intestine-specific genes in *Bmpr1a*^*Col1a2*^ mice (Fig. 4F, G). These findings suggest that *Bmpr1a* ablation in *Col1a2*^*+*^ stromal cells or *Acta2*^*+*^ myocytes/pericytes alone is sufficient to drive the development of invasive, intestinal-type-like gastric cancer, following preceding hyperplasia and polyposis. This is a surprising finding, because gastric cancer in mice usually requires combined deletion of multiple genes or treatment with mutagens together with *H. pylori* infection [23, 39].

In the corpus, the height of the glands remained largely unchanged, although a modest increase in the number of PCNA^+^ cells was observed (Supplementary Fig. S4A, B). qPCR indicated a modest upregulation of *Sox2* among the GSC markers tested, while the expression of differentiation markers was notably reduced (Supplementary Fig. S4C, D), suggesting a similar but lesser effect for stromal BMP signalling on the corpus GSCs.

### Loss of *Bmpr1a* in stromal cells causes DNA damage in GSCs

**Fig. 5:**
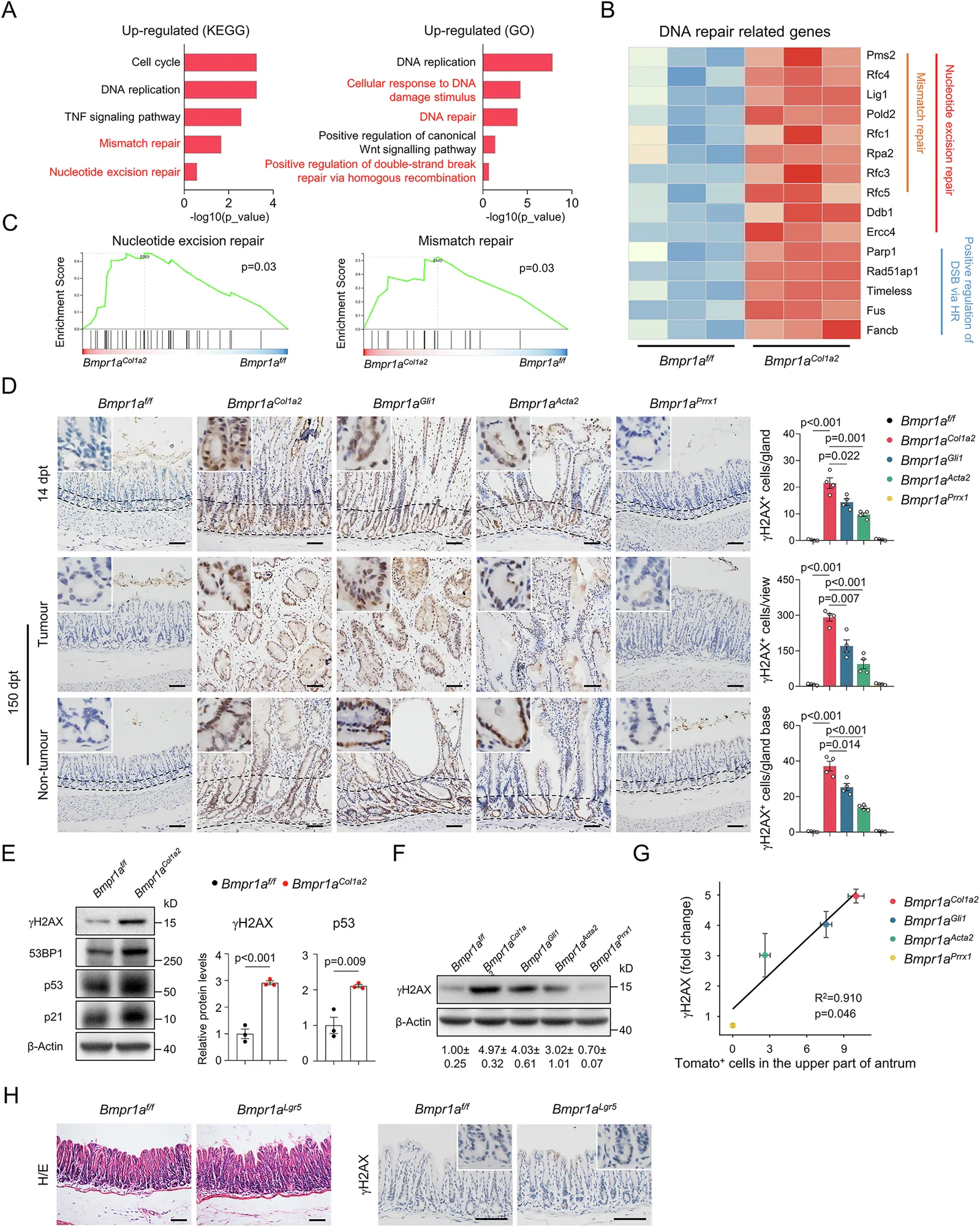
Loss of *Bmpr1a* in *Col1a2*^*+*^ or *Acta2*^*+*^ stromal cells causes DNA damage in GSCs. **A** KEGG and GO analyses of upregulated genes in the antral epithelia of *Bmpr1a*^*Col1a2*^ mice (Day 14 post TAM). *n* = 3 mice. **B** A heatmap displaying the upregulated DNA repair genes in antral epithelia of *Bmpr1a*^*Col1a2*^ mice. *n* = 3 mice. **C** GSEA confirmed the activation of DNA repair processes in the antral epithelia of *Bmpr1a*^*Col1a2*^ mice. **D** Representative γH2AX staining results from antrum sections of the four *Bmpr1a*-ablated mouse lines on Day 14 or 150 post TAM. Right panels: quantitation results. *n* = 4 mice for each genotype. **E** Western blot results showing levels of γH2AX and DNA damage response proteins in the antrum of *Bmpr1a*^*Col1a2*^ and control mice. Bottom panels: quantification data. *n* = 3 mice. The value for each band in the control group was set as 1.0 after normalization to β-Actin. Quantitation data for 53BP1 and p21 are shown in Supplementary Fig. S5D. **F** Western blot results for γH2AX in antrum samples of the four *Bmpr1a*-ablated mouse lines (Day 14 post TAM). The quantitative data are shown beneath the blot. *n* = 3 mice. **G** The correlation between the γH2AX levels and stromal cell abundance in the upper part of antrum (Fig. 5F). *n* = 3 mice for each genotype. **H** Representative H/E and γH2AX staining results of the antrum sections of *Bmpr1a*^*Lgr5*^ mice 5 months post TAM. Unpaired two-tailed Student’s *t* test was applied for (**D**) and (**E**). linear regression analysis was applied for (**G**). *p* < 0.05 was considered statistically significant. Scale bars: 100 μm.

How does stromal cell-specific ablation of *Bmpr1a* lead to invasive gastric cancer? GC development in mice typically requires simultaneous ablation of multiple genes or exposure to mutagens in conjugation with *H. pylori* infection [23, 39]. To understand how *Bmpr1a*^*Col1a2*^ mice develop invasive GC without other oncogenic stimuli, we isolated epithelial cells (EpCAM^+^CD45^-^CD31^-^CD51^-^) from the antrum of *Bmpr1a*^*Col1a2*^ and control mice (day 14 post-TAM) and conducted bulk RNA-seq (Supplementary Fig. S5A). Our analysis revealed upregulated expression of genes associated with the cell cycle, DNA replication, TNF pathway, Wnt pathway, DNA damage response, mismatch repair, nucleotide excision repair, and homologous recombination repair (Fig. 5A, B). Conversely, we observed downregulation of genes related to cell adhesion, epithelial differentiation, and gastric acid secretion (Supplementary Fig. S5B). Gene Set Enrichment Analysis (GSEA) further confirmed the activation of mismatch and nucleotide excision repair processes (Fig. 5C). These findings suggest that DNA damage has occurred in antral epithelia 14 days after *Bmpr1a* ablation in stromal cells. Immunostaining for γH2AX, a marker of double stranded DNA breaks (DSBs), revealed ongoing DNA damage in epithelial cells at the base 14 days after TAM, as well as in all epithelial cells in the tumours and at the base of the non-tumour regions 5 months post TAM, in *Bmpr1a*^*Col1a2*^ mice (Fig. 5D). γH2AX signals were detected solely in EpCAM^+^ epithelial cells (Supplementary Fig. S5C). Western blot analysis confirmed increases in γH2AX and DNA damage response proteins, including 53BP1, p53, and p21 in antral samples of *Bmpr1a*^*Col1a2*^ mice (Fig. 5E and Supplementary Fig. S5D), which may be induced to maintain genome fidelity in response to DNA damage [45]. We also sequenced the genomic DNA (the coding sequences) of antral cells of *Bmpr1a* ^*Col1a2*^ and control mice (14 days post-TAM) and uncovered significantly increased DNA lesions in *Bmpr1a*^*Col1a2*^ mice, including insertions, deletion, and point mutations (Supplementary Fig. S5E, F).

We also detected γH2AX signals in antral epithelia and GSCs of *Bmpr1a*^*Gli1*^ or *Bmpr1a*^*Acta2*^ mice (Fig. 5D). Western blot results confirmed an increase in γH2AX signals, which appeared to be positively correlated with the size of the stromal lineages in the upper part of antrum in these mice (Fig. 5F, G). These findings suggest that BMP-responding stromal cells collectively safeguard the genome of GSCs, explaining why *Bmpr1a*^*Col1a2*^ mouse line was the first to develops invasive GC. Intriguingly, no DNA damage was detected in the epithelial cells of the corpus across any of the four *Bmpr1a*-ablated mouse lines (Supplementary Fig. S5G), likely due to much fewer proliferating cells in the corpus compared to the antrum in *Bmpr1a*^*Col1a2*^ mice (Supplementary Figs. S3B, S4A).

We also generated *Bmpr1a*^*Lgr5*^ mice and found that *Bmpr1a* ablation in *Lgr5*^*+*^ GSCs caused mild hyperproliferation 5 months post TAM administration (Fig. 5H), consistent with a previous report that these mice do not develop invasive gastric tumours 8 months after *Bmpr1a* loss [46]. *Bmpr1a*^*Lgr5*^ mice showed no detectable DNA damage in the antrum (Fig. 5H), indicating that BMP signalling in stromal cells, rather than in GSCs themselves, is required to maintain GSC genomic stability and implicating stromal cell-derived factors in this process.

### Disruption of stromal BMP signalling also induces DNA damage in ISCs

We then examined the intestines due to similarities in stromal populations with the stomach. *Bmpr1a* ablation caused a modest increase in small intestine crypt height in *Bmpr1a*^*Gli1*^ mice, *Bmpr1a*^*Col1a2*^ mice, and *Bmpr1a*^*Acta2*^ mice at 150 days post TAM. Colon polyps formed in 100% of *Bmpr1a*^*Gli1*^ mice, 77.8% of *Bmpr1a*^*Col1a2*^ mice, and 37.5% of *Bmpr1a*^*Acta2*^ mice, though no invasive tumours were observed (Supplementary Fig. S6A, B). Polyp formation correlated with stromal population abundance, as our genetic tracing revealed that *Gli1*, *Col1a2*, *Acta2*, and *Prrx1* marked 50.7%, 36.4%, 24.4%, and 3.26% of Vimentin^+^ stromal cells, respectively (Supplementary Fig. S6C, D). Low levels of γH2AX signals were seen at crypt base in *Bmpr1a*^*Gli1*^ mice 14 days post-TAM (Supplementary Fig. S6E). At 150 days post-TAM, γH2AX was detected in all polyp cells and crypt bases of non-polyp areas in *Bmpr1a*^*Col1a2*^, *Bmpr1a*^*Gli1*^, and *Bmpr1a*^*Acta2*^ mice (Supplementary Fig. S6E). These results suggest disrupting stromal BMP signalling also induces DNA damage in ISCs, although to a lesser extent.

### Elevated Wnt/β-catenin signalling causes DNA damage and carcinogenesis in *Bmpr1a*-deficient mice

**Fig. 6:**
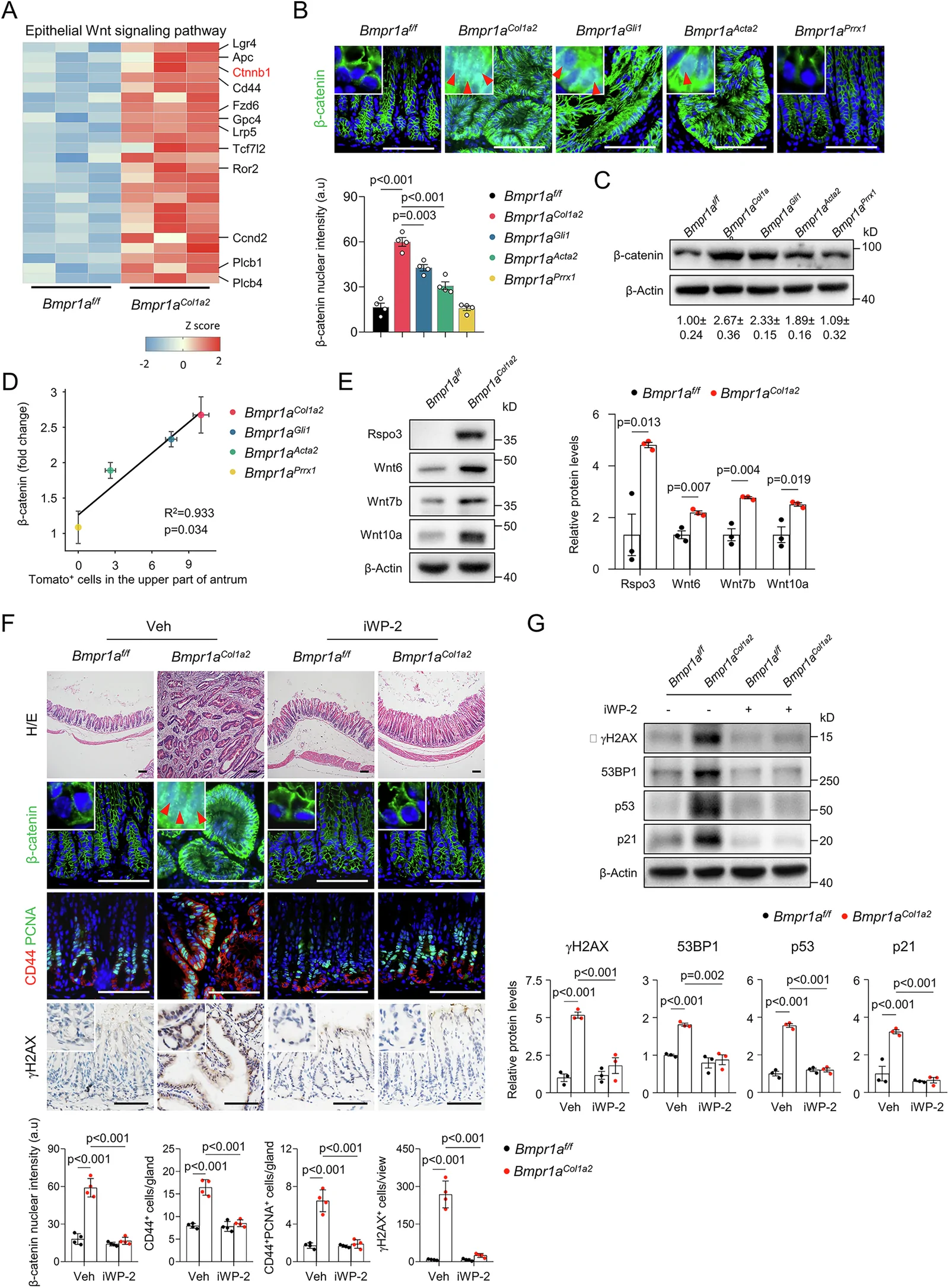
DNA damage and carcinogenesis in *Bmpr1a*^*Col1a2*^ mice requires Wnt/β-catenin signalling. **A** A heatmap displaying the expression of Wnt/β-catenin pathway genes in the antral epithelia of *Bmpr1a*^*Col1a2*^ and control mice. *n* = 3 mice. **B** Representative β-catenin immunostaining results for the antrum sections. Bottom panels: quantification data of nuclear β-catenin intensity. *n* = 4 mice for each genotype. **C** Western blot results showing protein levels of β-catenin in antrum samples from the four mouse lines. The quantitative data are shown beneath the blot. *n* = 3 mice. The value for each band in the control group was set as 1.0 after normalization to β-Actin. **D** The correlation between the β-catenin levels and stromal population size in the upper part of antrum (Fig. 6C). *n* = 3 mice for each genotype. **E** Western blot results showing elevated expression of Wnt ligands in the antrum samples of *Bmpr1a*^*Col1a2*^ mice. Bottom panel: quantitation data. *n* = 3 mice. **F** Representative H/E and β-catenin, CD44/PCNA or γH2AX immunostaining results showing the effects of iWP-2 (5 months’ treatment) on *Bmpr1a*^*Col1a2*^ mice. Antrum sections were stained with H/E or antibodies against PCNA and CD44 or γH2AX. Bottom panels: Quantitative data. *n* = 4 mice. **G** Western blot results showing the effects of iWP-2 on DNA damage and DNA damage response-related proteins in the antrum of *Bmpr1a*^*Col1a2*^ mice. Bottom panels: quantitation data. *n* = 3 mice. Unpaired two-tailed Student’s *t* test was applied for (**B**) and (**E**). Two-way ANOVA was applied for (**F**) and (**G**). linear regression analysis was applied for (**D**). *p* < 0.05 was considered statistically significant. Scale bars: 100 μm.

To investigate the cause of DNA damage in GSCs and epithelial cells, we reanalysed bulk RNA-seq data from epithelial cells. It revealed enhanced activation of Wnt/β-catenin signalling in the antral epithelia of *Bmpr1a*^*Col1a2*^ mice, alongside evidence of DNA damage (Figs. 5A and 6A). Wnts are the key pro-proliferative niche molecules for GSCs, which form a gradient that triggers a concentration-dependent response [47]. Immunostaining for β-catenin, which translocates into the nucleus upon stabilization in response to Wnts, revealed increased total and nuclear β-catenin signals in the antrum sections of *Bmpr1a*^*Col1a2*^ mice (Fig. 6B). Western blot analysis confirmed the elevated levels of β-catenin in antrum samples, which positively correlated with the size of the stromal populations in the upper part of antrum across all four mouse lines (Fig. 6C, D). Additionally, conditioned medium from stromal cells isolated from the antrum of *Bmpr1a*^*Col1a2*^ mice increased β-catenin levels in GES-1 gastric epithelial cells (Supplementary Fig. S7A). These results suggest that stromal *Bmpr1a* ablation enhances Wnt production and thereby boosts β-catenin signalling in GSCs or epithelial cells.

To investigate how stromal *Bmpr1a* ablation influences epithelial Wnt-β-catenin signalling, we performed bulk RNA-seq on Tomato^+^ stromal cells isolated from *Bmpr1a; Tomato*^*Col1a2*^ and *Tomato*^*Col1a2*^ mice. Our analysis revealed enriched expression of genes associated with cell cycle, gastric cancer, DNA replication, PI3K-Akt, mTOR, and positive regulation of Wnt signalling (Supplementary Fig. S7B). Conversely, we observed downregulation of genes related to fat digestion and absorption, lipid metabolism, and ATP synthesis (Supplementary Fig. S7C). While RNA-seq data indicated an increase in the expression of *Wnt5a*, *7b*, and *11* and *Rspo2*, albeit at low levels for *Wnt7b* and *Rspo2* (Supplementary Fig. S7D, E). Western blot confirmed a significant increase in Rspo3, Wnt6, Wnt7b and Wnt10a (Fig. 6E).

To assess the role of increased Wnt/β-catenin in carcinogenesis, we administered iWP-2, a widely used Porcn inhibitor that blocks Wnt secretion from stromal cells, thereby limiting Wnt ligand availability in the niche [48], to *Bmpr1a*^*Col1a2*^ mice concurrently with TAM administration. This effectively inhibited β-catenin activation in epithelial cells, reduced GSC hyperproliferation, and corrected the differentiation defects (Fig. 6F and Supplementary Fig. S8A). Additionally, hyperplasia in the corpus was prevented by iWP-2 (Supplementary Fig. S8B). iWP-2 also effectively prevented DNA damage, as evidenced by the absence of γH2AX signals on antral sections and reduced levels of γH2AX, 53BP1, p53, and p21 in antrum samples (Fig. 6F, G). These results suggest that high levels of Wnt molecules secreted from stromal cells not only promote GSC proliferation but also inflict DNA damage in GSCs, both of which may contribute to GC development.

### Wnt/β-catenin dose-dependently induces R-loop formation and DNA damage

**Fig. 7:**
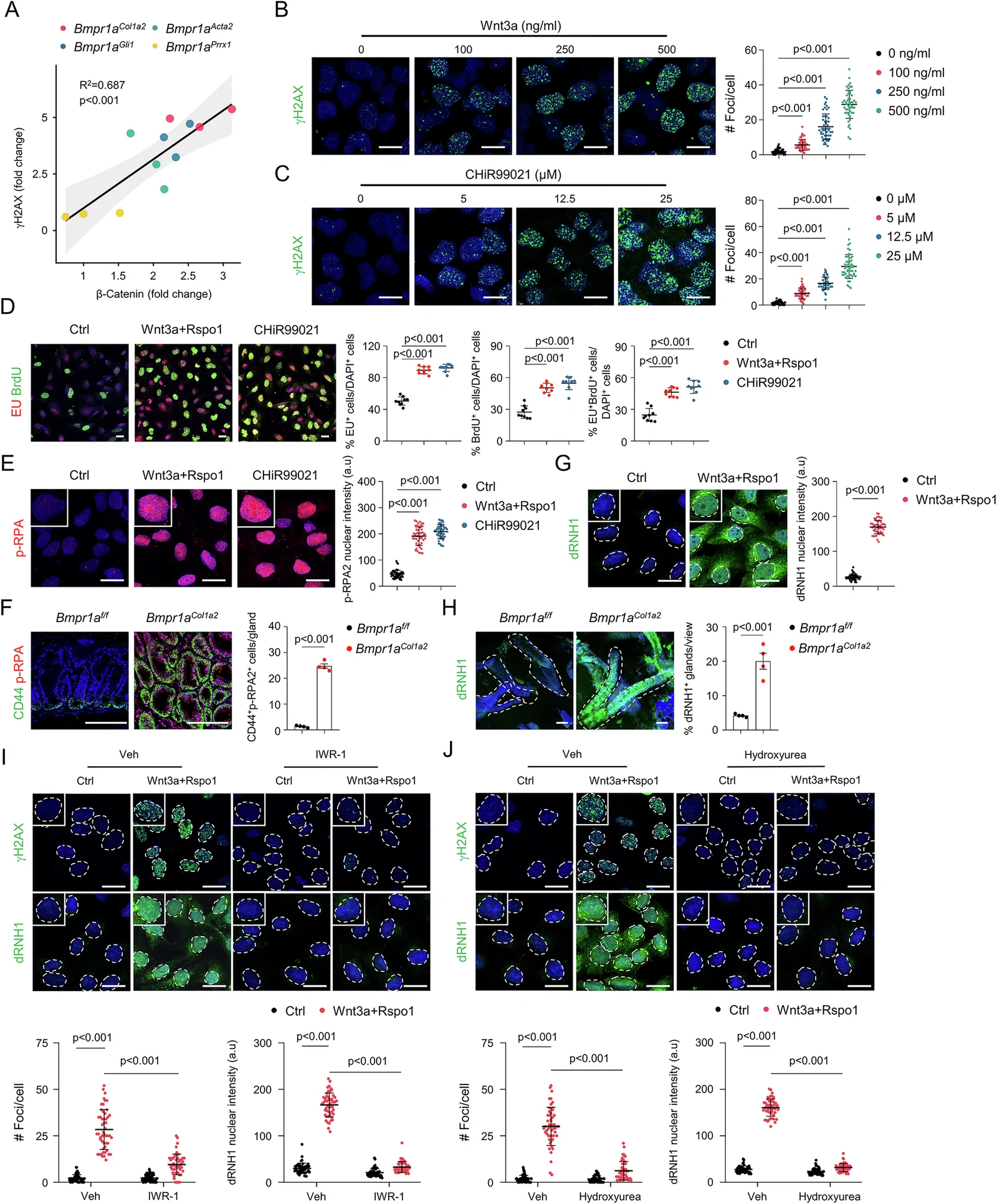
Hyperactive Wnt-β-catenin induces R-loop formation and DNA damage. **A** The correlation between the γH2AX levels (Fig. 5F) and the protein levels of β-catenin in the antrum (Fig. 6C). *n* = 3 mice for each genotype. **B** Wnt3a induced γH2AX^+^ foci formation in GES-1 cells in a dose-dependent manner after 12 h of treatment. Right panels: quantification data. *n* = 51 cells across 3 repeated experiments. **C** The Wnt agonist CHiR99021 induced γH2AX^+^ DNA damage foci formation in GES-1 cells in a dose-dependent manner after 12 h of treatment. Right panels: quantification data from 3 independent experiments. **D** Incorporation assays revealed increased transcription in S phase GES-1 cells in response to Wnt activation. Right panels: quantitation data. *n* = 9 (3 views each in 3 repeats). **E** Representative p-RPA staining results indicate that stalled DNA replication forks were increased by Wnt activation in GES-1 cells. Right panels: quantification data of nuclear p-RPA intensity from 3 independent experiments. **F** Representative CD44 and p-RPA staining results for the antrum of *Bmpr1a*^*Col1a2*^ and control mice. Right panels: quantification data of CD44^+^p-RPA^+^ cells per gland. *n* = 4 mice. **G** Wnt3a, in combination with Rspo1, induced R-loop formation in GES-1 cells after 12 h of treatment. Right panels: quantification data of nuclear dRNH1 intensity from 3 independent experiments. **H** Representative immunostaining results showing R-loop formation in isolated antral glands from *Bmpr1a*^*Col1a2*^ mice (14 days post TAM). Right panels: quantification data of the percentage of dRNH1^+^ gland. *n* = 4 mice. **I** IWR-1 inhibited DNA damage foci and R-loop formation in GES-1 cells. The cells were co-treated with Wnt3a/Rspo1 and IWR-1 for 12 h. Bottom panels: quantification data from 3 repeated experiments. **J** A low dose of hydroxyurea (HU, 0.01 mM) inhibited DNA damage foci formation and R-loop formation in GES-1 cells. The cells were treated with HU for 12 h, followed by Wnt3a/Rspo1 treatment for another 12 h. Bottom panels: quantification data from 3 repeated experiments. Unpaired two-tailed Student’s *t* test was applied for (**B**–**H**). Two-way ANOVA was applied for (**I**) and (**J**). Linear regression analysis was applied for (**A**). *p* < 0.05 was considered statistically significant. Scale bars: 100 μm for (**F**), 300 μm for (**B**)–(**E**), (**G**)–(**J**).

The genomes of tissue stem cells are protected by multiple mechanisms, including stem cell quiescence, accelerated DNA repair, and apoptosis [3, 4]. Our findings indicate that the levels of γH2AX were positively correlated with β-catenin levels in the antrum of all four *Bmpr1a*-ablated mouse lines (Fig. 7A), suggesting that β-catenin may play a role in DNA damage. To test this, we examined the effects of Wnt7b, BMP2, and EGF on DNA damage. In the gastric epithelial cell line GES-1, high doses of Wnt7b induced the formation of DNA damage foci, whereas neither BMP2 nor EGF did (Supplementary Fig. S9A–C). We confirmed this using another canonical Wnt ligand Wnt3a, commonly used in gastric organoid culture, and the canonical Wnt pathway agonist CHiR99021; both produced comparable DNA-damaging effects (Fig. 7B, C). Combinations of Wnt3a with Rspo1 or of Wnt7b with Rspo3 exhibited synergistic effects, leading to approximately 1.8 folds’ increase in DNA damage foci formation, whereas Rspo1 or Rspo3 alone induced minimal foci (Supplementary Fig. S9D). However, β-catenin was not localized to the foci (Supplementary Fig. S9E). Furthermore, ectopically expressed β-catenin also induced foci formation although β-catenin was not localized at the foci (Supplementary Fig. S9F, G). Similar experiments conducted with gastric cancer line NCI-N87 yielded comparable results (Supplementary Fig. S9H). These findings suggest that high levels of the canonical Wnt/β-catenin signalling can induce DSBs in a dose-dependent manner.

β-catenin is a transcription factor known for its pro-mitogenic activity [49]. Bulk RNA-seq data revealed enriched expression of genes involved in DNA replication and transcriptional misregulation in epithelial cells (Supplementary Fig. S10A). Simultaneous DNA replication and transcription can lead to transcription-replication collisions, the formation of R-loop (a three-stranded DNA-RNA hybrid structure), and subsequent genomic instability [50]. Indeed, we observed an increase in PCNA^+^ S phase antral epithelia in *Bmpr1a*^*Col1a2*^ mice (Supplementary Fig. S3B). Furthermore, Wnt3a in combination with Rspo1 or a Wnt agonist increased transcription in S phase cells (by 90%), as evidenced by increased ethyluridine incorporation in BrdU^+^ cells in transient labelling experiments (Fig. 7D). Additionally, we detected strong p-RPA signals in antral GSCs of *Bmpr1a*^*Col1a2*^ mice or GES-1 cells when Wnt/β-catenin was activated (Fig. 7E, F). RPA is a single-strand DNA-binding protein that interacts with RNase H1 and colocalizes with R-loops in cells. We also observed elevated expression of RNase H1, H2a, and H2b, enzymes that resolve R-loops (Supplementary Fig. S10A). These results suggest the formation of R-loops.

To confirm this, we used S9.6 or dRNH1 to stain for R-loops and detected increased signals in cells treated with Wnt3a plus Rspo1 or a Wnt agonist (Fig. 7G and Supplementary Fig. S10B). Consistently, antral glands isolated from *Bmpr1a*^*Col1a2*^ mice also showed evidence of R-loop formation (Fig. 7H). To corroborate the effects of Wnt/β-catenin signalling on R-loop formation and DNA damage, we treated GES-1 cells with IWR-1, an inhibitor of β-catenin activity. Immunostaining showed that Wnt3a- and Rspo1-induced R-loop formation and DNA damage were suppressed (Fig. 7I). Moreover, inhibiting DNA replication with a low dose of hydroxyurea blocked Wnt-induced DNA damage and R-loop accumulation (Fig. 7J and Supplementary Fig. S10C). These results suggest that highly activated β-catenin promotes R-loop formation by simultaneously increasing DNA replication and transcription.

### Dysregulation of the BMP-Wnt axis contributes to GC progression in humans

**Fig. 8:**
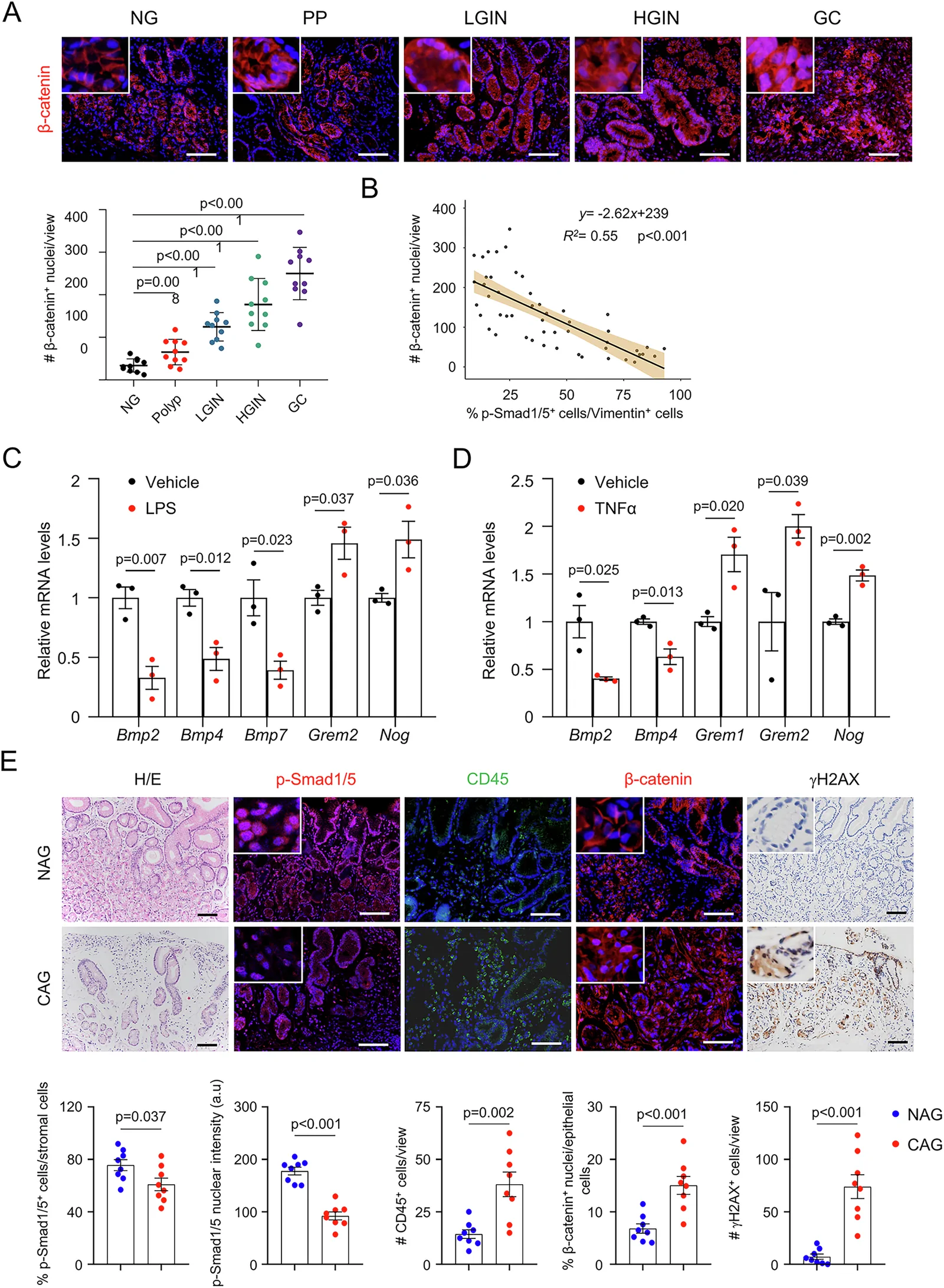
Chronic atrophic gastritis patient samples showed reduced BMP signalling and DSBs. **A** Immunostaining for β-catenin in patient sample sections. Bottom panels: quantification data. *n* = 10 per stage. **B** Analysis of the correlation between epithelial Wnt/β-catenin signalling and stromal BMP signalling in patient samples. *n* = 50. **C** qPCR analysis of the levels of BMP ligands and antagonists in gastric stromal cells treated with LPS. *n* = 3. **D** qPCR analysis of the levels of BMP ligands and antagonists in gastric stromal cells treated with TNFα. *n* = 3. **E** Representative H/E and immunostaining results for p-Smad1/5, CD45, β-catenin, and γH2AX in chronic atrophic gastritis patient samples. Bottom panels: Quantification data. *n* = 8. Unpaired two-tailed Student’s *t* test was applied for (**A**), (**C**), (**D**) and (**E**). Pearson’s correlation analysis was applied for (**B**). *p* < 0.05 was considered statistically significant.

The above findings indicate that a BMP-Wnt axis plays a critical role in suppressing gastric cancer initiation in mouse models. To assess the relevance of this axis in patients, we analysed TCGA gastric tumour datasets and observed reduced expression of the BMP target genes *ID1*, *ID3*, and *ID4* in GC samples (Supplementary Fig. S11A). This was accompanied by increased expression of Wnt pathway genes (*CTNNB1*, *WNT2*, *WNT3*, *WNT5a*, *WNT8b* and *WNT10a*) (Supplementary Fig. S11B). Importantly, higher *ID1* expression correlated with better prognosis, whereas higher expression of *CTNNB1*, *WNT8B*, and *WNT10A* correlated with poorer prognosis (Supplementary Fig. S11C). Furthermore, DNA damage and repair genes induced in the antral epithelium of *Bmpr1a*^*Col1a2*^ mice were also highly expressed in patient tumours (Supplementary Fig. S11D). Immunostaining of patient samples revealed that β-catenin-positive epithelial cells increased during gastric cancer progression (Fig. 8A), accompanied by a reduction in BMP-Smad1/5-positive cells (Fig. 1E). Stromal BMP signalling was negatively correlated with epithelial Wnt/β-catenin signalling (Fig. 8B). Together, these findings suggest that gastric cancer developing in *Bmpr1a*^*Col1a2*^ mice shares key molecular features with human gastric cancer.

### Inflammation suppresses BMP ligand expression in stromal cells

The primary risk factor for GC is *H. pylori* infection, which promotes cancer development through chronic inflammation and the generation of reactive oxygen species. We then asked what disrupts BMP/Smad signalling in stromal cells in gastric tumours and cancer samples. scRNA-seq analysis of GC samples provided a clue, revealing activation of immune response pathways in stromal cells, particularly TNF signalling (Fig. 1A). Immunostaining confirmed immune-cell infiltration in GC tumours (Supplementary Fig. S12A). Together with previous work showing that *H. pylori* infection suppresses BMP2 expression via T cell-derived IFNγ in mouse gastric glands [29], these observations suggested that inflammation suppresses BMP ligand expression. To test this, we treated primary stromal cells isolated from the mouse antrum with LPS, IFNγ, IL-6, or TNFα. qPCR analysis showed that these inflammatory stimuli reduced expression of *BMP2*, *BMP4*, and/or *BMP7* and increased expression of BMP antagonists *Grem1*, *Grem2*, and/or *Nog* in gastric stromal cells (Fig. 8C, D and Supplementary Fig.S12B, C). However, LPS did not alter expression of these BMP ligands or antagonists in gastric epithelial cells (Supplementary Fig. S12D), in line with the scRNA-seq results (Fig. 1C, D). These results suggest that inflammation inhibits BMP signalling primarily in stromal cells, through an autocrine mechanism.

### CAG patient samples and *H. pylori*-infected mouse samples showed reduced BMP signalling and increased DNA damage

Inflammation is a major feature of chronic atrophic gastritis, an early lesion caused by *H. pylori* infection that can persist even after bacterial eradication. We collected patient samples with nonmetastatic CAG (*H. pylori*-negative) and non-atrophic gastritis (NAG) as controls. Immunostaining showed that CAG samples had a lower percentage of p-Smad1/5-positive stromal cells and reduced nuclear p-Smad1/5 intensity compared with NAG, accompanied by an increase in CD45^+^ immune cells (Fig. 8E). CAG samples also exhibited a higher percentage of β-catenin-positive epithelial cells and stronger nuclear β-catenin staining (Fig. 8E). Moreover, γH2AX immunostaining revealed abundant DNA damage in epithelial cells from CAG samples, whereas NAG samples showed no detectable γH2AX signal (Fig. 8E). These findings indicate that DNA damage arises as early as the nonmetastatic atrophic gastritis stage after *H. pylori* infection, in a manner expected to drive gastric cancer initiation.

To strengthen the clinical relevance, we also generated an *H. pylori*-induced chronic gastritis mouse model. Immunostaining showed reduced nuclear p-Smad1/5 intensity, accompanied by increased CD45^+^ immune cells, nuclear β-catenin intensity and DNA damage in *H. pylori*-infected antral sections (Supplementary Fig. S12E). These results suggest that CAG in both mice and humans is associated with reduced BMP signalling and increased DNA damage.

## Discussion

Gastric cancer is driven by mutations in multiple driver genes, with *H. pylori* infection as the major risk factor. However, how this infection induces DNA damage and promotes GC development remains incompletely understood, particularly given the long latency between infection and cancer onset and the occurrence of GC even after bacterial eradication [20, 21]. Whereas previous studies have focused on virulence factors and reactive oxygen species generated by immune cells after *H. pylori* infection, our study suggests that chronic inflammation, from chronic atrophic gastritis through to gastric cancer progression, induces DNA damage in GSCs via disrupting stromal BMP signalling and overactivating Wnt/β-catenin signalling in GSCs, thereby initiating gastric cancer development. This contrasts with disruption of BMP signalling in GSCs, which does not induce DNA damage or gastric cancer development (as shown in previous studies and in this study [29, 46]). These findings reveal an unexpected mechanism by which *H. pylori* infection promotes somatic mutations and gastric cancer development by disrupting niche-GSC communication, beginning in the early stage of chronic atrophic gastritis.

The BMP gradient is well characterized in both gastric glands and intestinal crypts [29–31]. In the antrum, the stromal niche at the gland base, where BMP levels are low, continuously produces Wnt/R-spondin ligands, such as myocytes-derived Rspo3, to sustain GSC proliferation [29, 51]. Our data show that *Col1a2*^*+*^ stromal cells and *Acta2*^*+*^ myocytes/pericytes in the BMP-rich upper gland region are restrained from expressing Wnt/R-spondin molecules. When stromal BMP signalling is disrupted, for example by chronic inflammation, these cells overproduce Wnt/R-spondin ligands, which can diffuse toward the gland base and overwhelm normal negative-feedback regulation of Wnt signalling [49, 51, 52], leading to DNA damage in GSCs and ISCs. These findings uncover a previously unrecognized function for stromal cells located distal to the stem cell compartment, beyond their established role in extracellular matrix production [53]. They also suggest that the BMP gradient is required to maintain the Wnt gradient, providing a rapid mechanism to control niche signalling.

This study identifies a genotoxic consequence of excessively activated Wnt/β-catenin signalling. In our mouse models, the size of *Bmpr1a*-deficient stromal lineages in antral glands correlates with the level of Wnt signalling and the extent of DNA damage in GSCs. In vitro, Wnt ligands or agonists induce DNA damage in a dose-dependent manner. Mechanistically, overproduced Wnt/R-spondin ligands drive DNA damage in GSCs by simultaneously enhancing DNA replication and transcription and promoting R-loop formation, an effect that can be blocked by inhibiting Wnt secretion. The genotoxic effect for overactivated Wnt/β-catenin is further supported by a recent study in a β-catenin-driven T cell leukaemia model, which showed that β-catenin impairs homologous recombination DNA repair through Tcf-1, leading to genome instability, and another study showing that oncogenic β-catenin promotes lethality in BRCA1/2-deficient HeLa cells by promoting R-loop formation induced by BRCA1/2 deficiency; however, the effects in normal cells have not been reported [54, 55]. Thus, while somatic mutations in tissue stem cells are typically attributed to random replication errors or exogenous mutagens [1, 56], our work demonstrates that appropriate niche signalling is essential for preserving GSC genome stability, providing a conceptual bridge between tissue organization field theory and the somatic mutation theory of cancer origin [56].

Furthermore, our findings suggest a key role for inflammation in linking *H. pylori* infection to somatic mutations in GSCs. Inflammatory stimuli such as LPS and cytokines TNF, IL-6, and IFNγ suppress BMP ligand expression while inducing BMP antagonists. Biopsies from patients with chronic atrophic gastritis, a state of persistent inflammation, show reduced BMP signalling accompanied by DNA damage in epithelial cells. Gastric tumours likewise contain inflammatory infiltrates together with suppressed BMP signalling and DNA damage. Collectively, these data reveal a previously unrecognized mechanism by which chronic inflammation drives somatic mutations in gastrointestinal stem cells, in addition to the classical ROS-induced DNA damage pathway.

Overall, this study identifies a novel mechanism by which gastric stem cells acquire somatic mutations and undergo malignant transformation. Besides *H. pylori* infection, tissue ulceration or injury, for example, that induced by alcohol consumption, can also trigger inflammation. Inflammation disrupts niche-GSC communication and induces genotoxic stress in gastric stem cells, thereby promoting gastric cancer initiation. Furthermore, these findings suggest that pharmacologic inhibition of Wnt signalling, for example with agents such as iWP-2, could help prevent gastric cancer initiation in patients with atrophic gastritis by limiting mutagenesis.

## Materials and methods

### Mouse lines

The mouse experiments were conducted in accordance with the guidelines set forth by the National Research Council’s Guide for the Care and Use of Laboratory Animals. All experimental protocols were approved by the Institutional Animal Care and Use Committee of Shanghai Jiao Tong University, China [SYXK(SH)2011-0112, A2024014 and 2024132001]. The *Prrx1-CreERT* mouse line was sourced from Shanghai Biomodel Organism Science & Technology Development Co., Ltd [57]. The *Col1a2-CreERT*, *Lgr5-CreERT*, *Gli1-CreERT*, and *Rosa26-tdTomato* mouse lines were obtained from the Jackson Laboratory. The *Acta2-CreERT* mouse line was kindly provided by Gang Ma. The *Bmpr1a*^*f/f*^ mouse line was generated in Yuji Mishina’s laboratory [58]. *Prrx1-CreERT*, *Col1a2-CreERT*, *Lgr5-CreERT*, *Gli1-CreERT*, *Acta2-CreERT* and *Rosa26-tdTomato* mouse lines were C57BL/6-enriched background, while *Bmpr1a*^*f/f*^ mouse line was 129S7 background. We crossed *Bmpr1a*^*f/f*^ mouse line with *Col1a2-CreERT*, *Gli1-CreERT*, *Acta2-CreERT*, *Prrx1-CreERT* and *Lgr5-CreERT* mouse lines to generate *Bmpr1a*^*Col1a2*^, *Bmpr1a*^*Gli1*^, *Bmpr1a*^*Acta2*^, *Bmpr1a*^*Prr1*^and *Bmpr1a*^*Lgr5*^ mice. For genetic tracing experiments, we generate *Tomato*^*Col1a2*^, *Tomato*^*Gli1*^, *Bmpr1a*^*Acta2*^ and *Tomato*^*Prr1*^ mice. All in vivo experiments were done using 3-month-old male mice.

### Chronic gastritis mice model

Eight-week-old male C57BL/6J mice were used for this study. Mice were intragastrically infected with 10^8^ colony-forming units of *H. pylori* strain PMSS1 and sacrificed after 1 month. Gastric tissues were sectioned and subjected to immunostaining for p-Smad1/5, CD45, β-catenin, and γH2AX.

### Genetic tracing experiments

At 3 months of age, *Tomato*^*Col1a2*^, *Tomato*^*Gli1*^, *Tomato*^*Acta2*^, and *Tomato*^*Prrx1*^ mice were administered three intraperitoneal doses of tamoxifen (10 mg/kg). The mice were sacrificed 30 days post-tamoxifen (30 dpt), and their gastrointestinal tracts were harvested, fixed, sectioned, and stained with DAPI.

### Treatment with inhibitors

Male *Bmpr1a*^*f/f*^ and *Bmpr1a*^*Col1a2*^ mice were randomly assigned to four groups: untreated *Bmpr1a*^*f/f*^ (vehicle), treated *Bmpr1a*^*f/f*^ (iWP-2), untreated *Bmpr1a*^*Col1a2*^ (vehicle) and treated *Bmpr1a*^*Col1a2*^ (iWP-2). Randomization was performed by a researcher not involved in the experiment. For in vivo Wnt signaling inhibition, mice were orally gavaged with iWP-2 (5 mg/kg, MCE, #HY-13912) every other day for 5 months, also starting at the time of TAM administration. iWP-2 were prepared according to the manufacturer’s instructions.

### Human CAG and gastric tumour samples

Normal human gastric tissue (NG, *n* = 10), NAG (Non-atrophic gastritis, *n* = 8), CAG (Chronic atrophic gastritis, *n* = 8), polypus (PP, *n* = 10), low-grade intraepithelial neoplasia (LGIN, *n* = 20), high-grade intraepithelial neoplasia (HGIN, *n* = 15), and gastric cancer (GC, *n* = 15) samples were obtained from the Department of Gastroenterology, Ruijin Hospital, China. The study protocol was approved by the Research Ethics Committee of Ruijin Hospital (Approval NO. 2019-95). Patient information is summarized in Supplementary Table S1. Written informed consent was obtained from all participants.

### Haematoxylin/eosin staining and immunostaining

The mice were sacrificed, and the tissues were fixed in 4% paraformaldehyde overnight. Following dehydration in alcohol and embedding in paraffin, samples from the stomach, intestine and colon were sectioned longitudinally at 4 μm. The sections were stained with hematoxylin and eosin (H&E) or alcian blue according to a standard protocol. For immunofluorescence staining, tissue slides were permeabilized with 1% Triton X-100 in PBS for 15 min. For antigen retrieval, the slides were boiled at 99 °C in citrate buffer or EDTA buffer (pH = 8.0). After cooling, the slides were blocked with 20% goat serum in PBS for 30 min at room temperature and incubated with primary antibodies (1:100) overnight at 4 °C. The primary antibodies used included Ki67 (Invitrogen, MA5-14520), PCNA (Santa Cruz, sc-56), gastrin (DAKO, A0568), H^+^K^+^ATPase (Santa Cruz, sc-374094), AQP5 (Invitrogen, PA5-36529), Sox9 (Millipore, ab5535), CD44 (Santa Cruz, sc-18849), CD45 (Abcam, ab10558), CD31 (Abcam, ab56299), Vimentin (Abcam, ab92547), β-catenin (CST, 8480), EpCAM (Abcam, ab221552), αSMA (Abcam, ab5694), Gli1 (Invitrogen, MA5-32553), p-Smad1/5 (CST, 9516) and γH2AX (Abcam, ab81299). The following day, the slides were rewarmed at 37 °C for 1 h, washed in PBS, and incubated with secondary antibodies at 37 °C for 1 h. The results were visualized using a Nikon imaging system (ECILPSE, 80i) or a laser confocal imaging system (Leica TCS SP8).

For immunohistochemical staining, the paraffin sections were dewaxed and rehydrated. The samples were treated with 3% hydrogen peroxide (H₂O₂) for 20 min to block endogenous peroxidase activity and then permeabilized with 0.1% Triton X-100 in PBS for 30 min. Antigen retrieval was performed by heating the samples in sodium citrate solution at 99 °C for 30 min. The samples were then blocked and incubated with primary antibodies (γH2AX (Abcam, ab81299)) and secondary antibodies conjugated to horseradish peroxidase (HRP). Subsequently, the samples were incubated with a DAB stain kit (Boster), washed, and treated with SABC solution at 37 °C for 1 h in the dark. Finally, the slides were dehydrated through two changes of 95% alcohol and absolute alcohol, cleared in two changes of xylene, and mounted with resin for observation.

### Quantification of the nuclear fluorescence intensity

ImageJ was used to quantify nuclear p-Smad1/5, β-catenin, p-RPA, and dRNH1 signal intensity. For p-Smad1/5, blinded investigators measured the mean nuclear fluorescence intensity in stromal cells from human NAG and CAG samples (≥30 cells per sample). Background was subtracted using signal from non-nuclear p-Smad1/5-stained regions within the same sample. Quantification of p-RPA and dRNH1 signal intensity in GES-1 cells, and β-catenin signal intensity in *Bmpr1a*-ablated mice, was performed using the same procedure.

### Primary gastric stromal cell isolation and culture

*Tomato*^*Col1a2*^ mice and *Bmpr1a; Tomato*^*Col1a2*^ mice were sacrificed 30 days following tamoxifen treatment. The antrum of these mice was collected and washed three times with cold PBS. The tissues were then minced into small pieces (1 mm) and digested in RPMI 1640 medium containing collagenase II (3 mg/ml) and dispase II (3 mg/ml) at 37 °C for 1 h. After filtration and centrifugation, Tomato^+^ gastric stromal cells were sorted using a Bio-Rad S3e sorter. These cells were either used for RNA sequencing or cultured in MEM Alpha medium supplemented with 10% fetal bovine serum and 1% penicillin‒streptomycin at 37 °C in a 5% CO₂ atmosphere.

For inflammatory stimulation, gastric stromal cells were treated with LPS (5 ng/ml, Selleck, S7850), IFNγ (2 ng/ml, Peprotech, 315-05-20), TNFα (10 ng/ml, Peprotech, 300-01A-50), or IL-6 (5 ng/ml, Peprotech, 216-16-10) for 12 h, after which the cells were collected for qPCR analysis.

### Gastric organoid culture

The antrum from normal mice was harvested, and the middle portion was opened. The tissues were rinsed several times in cold PBS containing 1% penicillin‒streptomycin to thoroughly remove food residues and other impurities. The gastric epithelial layer was then separated from the muscle layer under a stereomicroscope. The epithelial layer was cut into 1-mm fragments and digested in 2.5 mM EDTA/DPBS at 4 °C for 1 h. The gland fragments were filtered through a 70 µm strainer (Thermo Fisher), washed with cold DPBS, and centrifuged at 1000 rpm for 5 min. The resulting gland pellets were suspended in growth factor-reduced (GFR) phenol-free Matrigel (Corning) and seeded in 48-well plates in 35 µl drops. The glands were cultured at 37 °C for 10 min to solidify and then cultured in advanced DMEM/F12 medium (Gibco) supplemented with HEPES (Gibco, 10 mM), L-GlutaMax (Gibco, 1X), B27 (Gibco, 1X), N2 (Gibco, 1X), N-acetyl-L-cysteine (Sigma, 1 mM), R-Spondin1 (RD, 1 µg/ml), EGF (Peprotech, 50 ng/ml), Noggin (Peprotech, 100 ng/ml), FGF10 (Peprotech, 100 ng/ml), Wnt3a (Peprotech, 100 ng/ml), and Penstrep (Gibco, 1X). The medium was changed every 2–3 days. At different time points (Days 1, 3, 5, and 7), the organoids were observed under an inverted microscope, and the organoid diameters were measured. For co-culture experiments, the organoids were mixed with normal or mutant gastric stromal cells in GFR Matrigel and distributed in 35 µl drops over a 48-well plate. After 7 days of co-culture, organoids were harvested to assess the expression of stem cell and differential markers.

### Flow cytometry analysis and sorting

To analyse the proportions of *Col1a2*, *Gli1*, *Acta2*, and *Prrx1* lineage stromal cells (Tomato^+^CD51^+^Epcam^-^CD31^-^CD45^-^) within the total stromal cell population (CD51^+^Epcam^-^CD31^-^CD45^-^), *Tomato*^*Col1a2*^, *Tomato*^*Gli1*^, *Tomato*^*Acta2*^, and *Tomato*^Prrx1^ mice (*n* = 3 for each mouse line) were intraperitoneally injected with three doses of tamoxifen at 3 months of age and sacrificed 30 days after tamoxifen treatment. After removal of the muscle layer, the antral epithelial layer was digested with collagenase II and dispase II. Following filtration and centrifugation, the cells were stained with the following fluorescence-conjugated antibodies: anti-CD31-FITC (102406, Biolegend), anti-CD45-FITC (103108, Biolegend), anti-CD51-BV421 (740062, BD Biosciences), and anti-Epcam-PE-Cy7 (118216, Biolegend). The corresponding IgG isotypes served as negative controls, with gating thresholds determined by isotype-stained controls. Flow cytometry analyses were conducted using a Beckman Cytoflex S with CytExpert software (v2.1.0.92), and sorting was performed on a Bio-Rad S3e using ProSort software (v1.6.0.12). The FACS data were analysed with FlowJo (v10.5.3), and all figures were displayed in Biex mode.

### Cell line-based studies

The human gastric cancer cell line NCI-N87 was purchased from National Collection of Authenticated Cell Culture and the human gastric epithelial cell line GES-1 was kindly provided by Dr. Lizong Shen. Prior to the formal experiments, all cell lines were authenticated and tested negative for mycoplasma contamination. NCI-N87 was cultured in RPMI 1640 medium supplemented with 10% fetal bovine serum and 1% penicillin-streptomycin. The human gastric epithelial cell line GES-1 was cultured in DMEM supplemented with 10% fetal bovine serum and 1% penicillin‒streptomycin at 37 °C in a 5% CO₂ atmosphere. The medium was changed every 2–3 days. For immunostaining, GES-1 or NCI-N87 cells were fixed with cold 4% paraformaldehyde (PFA) for 15 min. After treatment with PBS containing 2% BSA and 0.5% Triton-X, the cells were incubated overnight at 4 °C with primary antibodies against p-RPA2 (Abcam, ab211287), β-catenin (CST, 8480), or γH2AX (Abcam, ab22551). The cells were then incubated with secondary antibodies for 1 h at 37 °C.

To assess DNA damage, GES-1 or NCI-N87 cells were treated with ChiR99021 (5–25 µM), Wnt3a/Rspo1 (Wnt3a: 100–500 ng/ml; Rspo1: 0.5 µg/ml), Wnt7b/Rspo3 (Wnt7b: 100–500 ng/ml; Rspo3: 0.5 µg/ml), BMP2 (100 ng/ml), or EGF (100 ng/ml) for 12 h, followed by immunostaining for γH2AX. To examine the effect of hydroxyurea (0.01 mM) on DNA damage, GES-1 cells were treated with hydroxyurea for 12 h before adding Wnt or the agonist. To assess the effect of IWR-1 (20 μM, Selleck, S7850) on DNA damage, GES-1 cells were co-treated with Wnt3a/Rspo1 and IWR-1 for 12 h.

For β-catenin overexpression, PT3-N90-β-catenin (#31785, Biobw) was transfected at varying doses (0.5–2.5 µg) using Lipofectamine™ 2000, following the standard procedure provided by Invitrogen.

To test the effect of stromal cell-secreted Wnt/Rspo molecules, GES-1 cells were maintained in DMEM supplemented with 50% conditioned medium from stromal cells derived from *Tomato*^*Col1a2*^ or *Bmpr1a; Tomato*^*Col1a2*^ mice for 12 h, and then harvested for Western blot analysis.

### R-loop detection

For dRNH1-GFP staining in cell culture, GES-1 cells were fixed with cold 4% paraformaldehyde (PFA) for 10 min, washed with PBS, and then permeabilized with PBS containing 0.3% Triton X-100 for 10 min at room temperature. After blocking with PBS containing 2% BSA and 0.2% Triton X-100 for 1 h at 37 °C, the GES-1 cells were incubated with 30 µl of dRNH1-GFP (0.24 mg/ml) (provided by Dr. Xiaolin Guo, Shanghai Jiao Tong University School of Medicine) for 1 h at 37 °C, as previously described [59].

For dRNH1-GFP staining in vivo, antral glands were isolated following the protocols for gastric organoid culture. After fixation, permeabilization, and blocking, the glands were mixed with PBS containing 50% Matrigel and spread onto slides for subsequent dRNH1-GFP staining.

For S9.6 immunostaining in cell cultures, GES-1 cells were fixed with ice-cold 100% methanol for 5 min at −20 °C and washed three times with PBS. For enzymatic digestion, RNase H (NEB) was diluted 1:50 in RNase H buffer and incubated with the cells for 3 h at 37 °C. After permeabilization and blocking, the GES-1 cells were incubated with S9.6 (Kerafast, ENH001) overnight at 4 °C. The cells were then incubated with secondary antibodies for 1 h at 37 °C.

### Ethyluridine (EU)/BrdU transient labelling experiment

To analyse transcriptional activity and DNA replication following Wnt activation, GES-1 cells were treated simultaneously with CHiR99021 (12.5 µM) or Wnt3a/Rspo1 (Wnt3a: 250 ng/ml; Rspo1: 0.5 µg/ml) and BrdU (3 µg/ml). After incubation for 2.5 h at 37 °C, 5-ethynyl uridine (EU) was added to the medium at a dilution of 1:1000 and incubated for an additional 30 min at 37 °C. The number of cells enriched with EU was determined according to the manufacturer’s protocol (Cell-Light EU Apollo567 RNA Imaging Kit, RiboBio), followed by incubation with a BrdU antibody (Sigma, B8434).

### Whole exome sequencing

The genomic DNA was extracted from the antrum of *Bmpr1a*^*Col1a2*^ and control mice (14 days post-TAM) and fragmented into 200–300 bp fragments through sonication, followed by end-repairing. Fragmented DNA was then subjected to exome capture using Agilent50M followed by next generation sequencing on the DNBSEQ platform. Sequence reads were mapped to the mouse reference genome assembly (mm10) using Burrows-Wheeler Aligner. SNP and INDEL were detected using the method of population site variation detection with the software GATK. Filtering and screening were further conducted according to quality value, depth, repeatability and other factors.

### Quantitative PCR

Tissues or cells were lysed, and total RNA was isolated using TRIzol reagent. cDNA was reverse transcribed from 1 µg of RNA using the PrimeScript RT Reagent Kit (TaKaRa, RR037A), and quantitative PCR was performed on a Roche LightCycler 480. The primer sequences used are listed in Supplementary Table S2 and have been previously reported in other studies (https://pga.mgh.harvard.edu/primerbank/).

### Western blot

Western blotting was performed following standard procedures. Proteins were extracted from the whole antrum or gastric stromal cells using RIPA lysis buffer supplemented with protease and phosphatase inhibitors. Equal amounts of total protein were subjected to SDS-PAGE and transferred to PVDF membranes (Millipore). The primary antibodies used included β-catenin (CST, 8480), Rspo3 (Invitrogen, PA5-100324), Wnt6 (Abcam, ab154144), Wnt7b (Invitrogen, PA5-103480), Wnt10a (Abcam, ab106522), β-Actin (Santa Cruz, sc-47778), γH2AX (Abcam, ab81299), 53BP1 (Abcam, ab21083), p53 (CST, 2524), and p21 (Santa Cruz, sc-817). Western blot results were visualized and quantified using the FluoChem M system (ProteinSimple) and ImageJ.

### RNA sequencing

FACS-sorted gastric stromal cells (Tomato^+^) and epithelial cells (Epcam^+^CD45^-^CD31^-^CD51^-^) from *Tomato*^*Col1a2*^ and *Bmpr1a; Tomato*^*Col1a2*^ mice were lysed, and total RNA was isolated (*n* = 3 per group). After quality control, cDNA was synthesized and amplified into DNA nanoballs (DNBs), which were sequenced using a BGI-500 system (BGI-Shenzhen, China). Gene expression levels were calculated and aligned using HISAT (v2.0.4), based on the Burrows-Wheeler transform and Ferragina–Manzini algorithms. Clean reads were assessed for randomness, coverage, and saturation. Differentially expressed genes were defined as those with statistically significant *p*-values (*p* < 0.05) and a fold change ≥1.5. For pathway and Gene Ontology (GO) analyses, candidate genes were assigned according to their official classification with KEGG or GO annotations, and functional enrichment was performed using the phyper function in R. Additionally, gene set enrichment analysis (GSEA) was conducted using GSEA_4.0.2 software (https://www.broadinstitute.org/gsea/), with parameters: metric = weighted, permutation = 1000, and metric = Signal2Noise. All bulk RNA-seq datasets have been deposited in public databases.

### Single-cell sequencing analysis

Single-cell sequencing of mouse corpus and antrum mesenchymal cells and patients with gastric cancer were subsets from GSE224737 and GSE183904. The generated gene-cell expression matrix was analysed using R version 3.6.1 and Seurat version 3.1.5. The following criteria were applied for cell inclusion: fewer than 200 expressed genes and more than 25 unique molecular identifiers (UMIs) mapped to mitochondria. Filtered cells were then subjected to downstream graph-based clustering. Differentially expressed genes (DEGs) in each cluster were identified using the “FindAllMarkers” function in Seurat. DEGs were mapped to the KEGG and GO databases, and enrichment analysis of GO terms or KEGG pathways was performed using the clusterProfiler R package, as previously described.

### Analysis of data from the Cancer Genome Atlas

TCGA datasets were utilized to analyse the relative mRNA levels of Wnt and BMP pathway genes. Transcriptome data from gastric cancer patients (NG = 36, GC = 410) and colorectal cancer (NC = 41, CRC = 465) were downloaded from https://portal.gdc.cancer.gov/. Unpaired Student’s *t* test was used to analyse differences between the normal and cancer groups, with a *p*-value < 0.05 indicating statistical significance. TCGA datasets were also employed to examine gene expression and overall survival (OS). TCGA integrates gene expression and clinical data, which are managed by a PostgreSQL server. The correlations between the expression of Wnt and BMP pathway genes and OS were analysed using an online resource (https://kmplot.com/), and the two patient cohorts were compared via a Kaplan–Meier survival plot.

### Statistical analysis

All data are presented as means ± standard deviations (SDs). Unpaired, two-sided Student’s *t* test (for comparisons between two groups) and two-way ANOVA with Bonferroni correction (for comparisons involving more than two groups) were conducted, as indicated in the fig. legends. Linear regression and pearson’s correlation analysis was performed to assess the relationship between variables. A *p*-value < 0.05 was considered statistically significant. No formal statistical methods were used to pre-determine sample size. The sample size was determined based on previous experience and ethical considerations and considered sufficient to detect the expected biological effect per group. The number of mice used in each experiment is specified in the fig. legend, and no data were excluded. Investigators were blinded to group allocation during animal experiments, including treatment administration and outcome assessment.

## Supplementary information

supplemental information

original western blot

## Data Availability

The raw data from bulk-RNA sequencing in this study have been deposited to the GEO database (https://www.ncbi.nlm.nih.gov/geo/) under accession number GSE286383 and GSE286027. The raw data of whole exome sequencing were deposited to the GSA database (https://ngdc.cncb.ac.cn/gsa/) under accession number CRA026877.
