# Supplementary material for: Stromal BMP signalling maintains stem cell genome integrity and prevents gastric cancer initiation
Source: Cell Death Dis. 2026 Jul 17;17(1):882. doi: 10.1038/s41419-026-09088-z (PMC13658785; doi:10.1038/s41419-026-09088-z)
Supplement: Supplementary file 1 [file 41419_2026_9088_MOESM1_ESM.pdf]

Supplementary Information for

Stromal BMP signalling maintains stem cell genome integrity and prevents gastric cancer initiation

Zihao Li[1,9], Zhengting Wang[2,9], Ruichen Yang[1,9], Jiarui Zhao[1], Shaoyang Zhang[1], Liuchun Feng[1], Haiyu Shen[1], Hua Yue[3], Zhenlin Zhang[3], Xiaolin Guo[4], Dengli Hong[4], Yuji Mishina[5], Ye-Guang Chen[6], Qiang Wu[7], Huijuan Liu[1,8,10], Baojie Li[1,8,10]

[1]Bio-X Institutes, Shanghai Jiao Tong University, Shanghai 200240, China.

[2]Department of Gastroenterology, Ruijin Hospital, School of Medicine, Shanghai Jiao Tong University, Shanghai, China.

[3]Department of Osteoporosis and Bone Diseases, Shanghai Clinical Research Center of Bone Diseases, Shanghai Jiao Tong University of Medicine Affiliated Sixth People's Hospital, Shanghai, China

[4]Institute for Translational Medicine on Cell Fate and Disease, Shanghai Ninth People's Hospital, Key Laboratory of Cell Differentiation and Apoptosis of Ministry of Education, Department of Pathophysiology, Shanghai Institute of Hematology, Ruijin Hospital, Shanghai Jiao Tong University School of Medicine, Shanghai, China.

[5]School of Dentistry, University of Michigan, Ann Arbor, MI 48109, USA.

[6]State Key Laboratory of Membrane Biology, Tsinghua-Peking Center for Life Sciences, School of Life Sciences, Tsinghua University, Beijing, 100084, China.

[7] The State Key Laboratory of Mechanism and Quality of Chinese Medicine, Macau University of Science and Technology. Avenida Wai Long, Taipa, Macau SAR, China.

[8]Shanghai Institute of Stem Cell Research and Clinical Translation, Shanghai 200120, China.

[9] Equal contributions

[10] Corresponding authors

Table S1 The patient sample information.

NO. of patient samples						
NG	NAG	CAG	PP	LGIN	HGIN	GC
G14730342	A502604E6	U01927818	2022-092717	2023-004648	2022-091493	2022-090406
13671749	H509FF716	D02879877	2022-094352	2023-006125	2023-056892	2022-091452
931101100006218	C11027070	D04095777	2022-094700	2023-006612	2023-049526	2022-095366
RJ012408140131	P10330224	C01241866	2022-095629	2023-007831	2023-045357	2022-096063
Z1271724	G5010E1F3	K01024536	2023-000906	2023-008629	2023-031322	2022-089522
F00484796	N01239280	E01941904	2023-005014	2023-009282	2023-004324	2022-089661
V18309458	F12104201	C0205072X	2023-009854	2023-011805	2023-002496	2023-056575
A0226108X	B01412074	U00530798	2023-011648	2023-012679	2023-044024	2023-006960
24384542x320			2023-013109	2023-014697	2023-030160	2023-060512
13611804331			2023-014466	2023-019311	2023-024955	2023-037893
				2023-020377	2023-017909	2023-037325
				2023-022308	2023-000926	2023-036550
				2023-023004	2024-078305	2023-034587
				2023-026512	2024-072729	2023-029326
				2023-031017	2024-069715	2024-065840
				2023-032164		
				2023-034237		
				2023-037410		
				2023-037447		
				2023-041136		

Table S2 Primers for qPCR.

gene	Primer-F (5'-3')	Primer-R (5'-3')
Gastrin	GGACAGGGACCAATGAGG	CCAAAGTCCATCCATCCGTAGG
Muc5ac	GCCGTGTCAGGAGTCTAATACC	AGCCTAGCCACCACCTTAG
Tff2	TGCTCTGGTAGAGGGCGAG	CGAGCTAGAGTCAAAGAG
Muc6	AGCCCACATTCCCTATCAGC	CACAGTGGAAGATTGCGAGAG
Sst	ACCGGGAAAAGGAACTGG	TTGCTGGGTTCGAGTTGGC
Neurog3	AGTGCTCAGTTCCAATTCCAC	CGGCTTCTTCGCTTTTTGCTG
Atp4a	GATGGAGATTAACGACCACCAG	ACGGGCAAACTTCACATACTC
Clca1	CTGTCTTCCTCTTGATCCTCCA	CGTGGTCTATGGCGATGACG
Cdx2	CAAGGACGTGAGCATGTATCC	GTAACCACCGTAGTCCGGGTA
Aqp5	TCCATGAACCCAGCCCGATCTT	GAAGTAGAGGATTGCAGCCAGG
Cd44	TCGATTTGAATGTAACCTGCCG	CAGTCCGGGAGATACTGTAGC
Cck2r	GCCGTTTCCTACCTCATGGGGGT	CCCTTGGTTTCGGACCCGGC
Sox9	GAGCCGGATCTGAAGAGGGA	GCTTGACGTGTGGCTTGTTC
Sox2	GGCAGCTACAGCATGATGCAGGAGC	CTGGTCATGGAGTTGTACTGCAGG
Lgr5	AGAGCCTGATACCATCTGCAAAC	TGAAGGTCGTCCACACTGTTGC
Mist1	GCTGACCGCCACCATACTTAC	TGTGTAGAGTAGCGTTGCAGG
Pgc	ATGAAGAGTATCCGGGAGACC	TGGGCTCATAGAGTACACTGTAG
Gif	GAAAAGTGGATCTGTGCTACTTGCT	AGACAATAAGGCCCCAGGATG
Lrig1	TTGAGGACTTGACGAATCTGC	CTTGTTGTGCTGCAAAAAGAGAG
Bmp2	GGGACCCGCTGTCTTCTAGT	TCAACTCAAATTCGCTGAGGAC
Bmp4	TTCCTGGTAACCGAATGCTGA	CCTGAATCTCGGCGACTTTTT
Bmp7	ACGGACAGGGCTTCTCCTAC	ATGGTGGTATCGAGGGTGGAA
Grem1	CTGGGGACCCTACTGCCAA	TTTGCACCAATCTCGCTTCAG
Grem2	GGTAGCTGAAACACGGAAGAA	TCTTGCACCAGTCACTCTTGA
Nog	GCCAGCACTATCTACACATCC	GCGTCTCGTTCAGATCCTTCTC
Gapdh	TGTGAACGGATTTGGCCGTA	ACTGTGCCGTTGAATTTGCC

Supplementary Figures

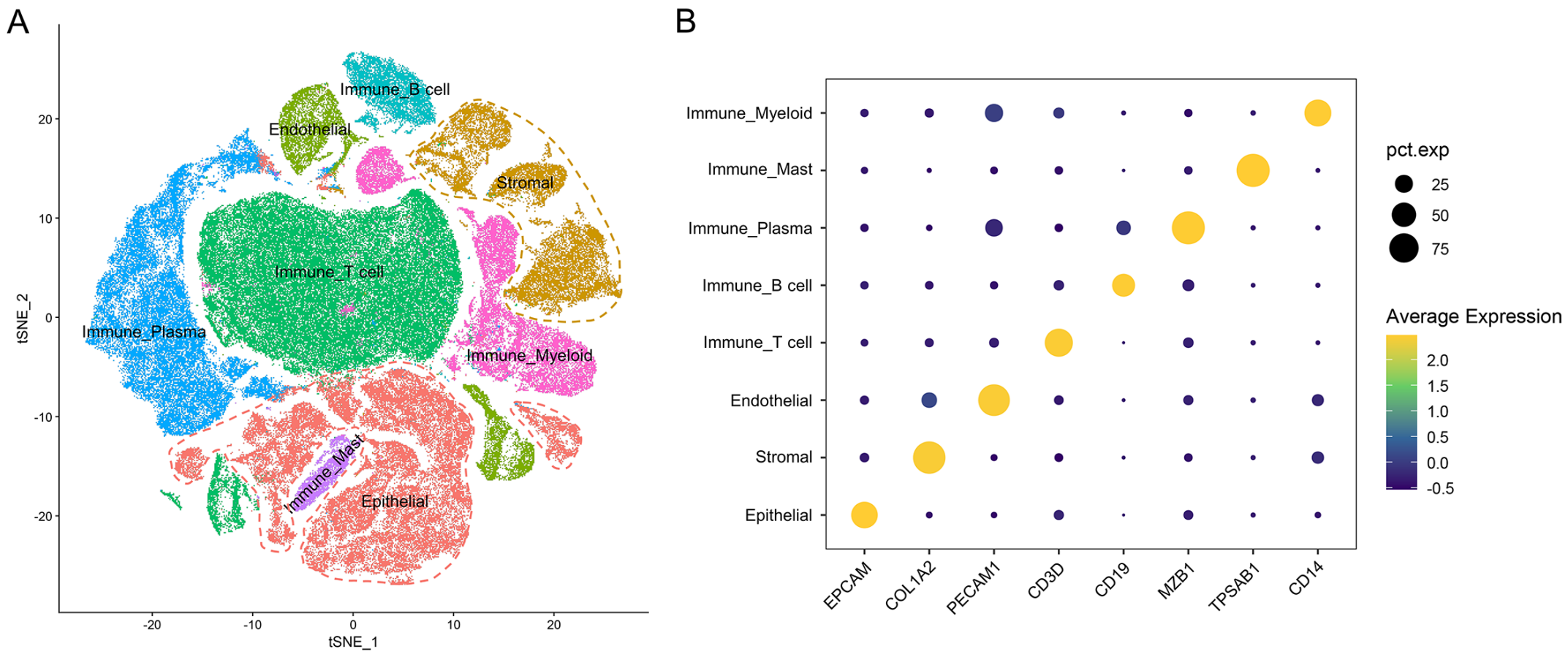

Figure S1. scRNA-seq analysis of human gastric cancer cells.

A. Clustering of cells of human gastric cancer samples (GSE183904) via scRNA-seq analysis. NG=11, GC=29.

B. Markers used to cluster the 8 distinct populations.

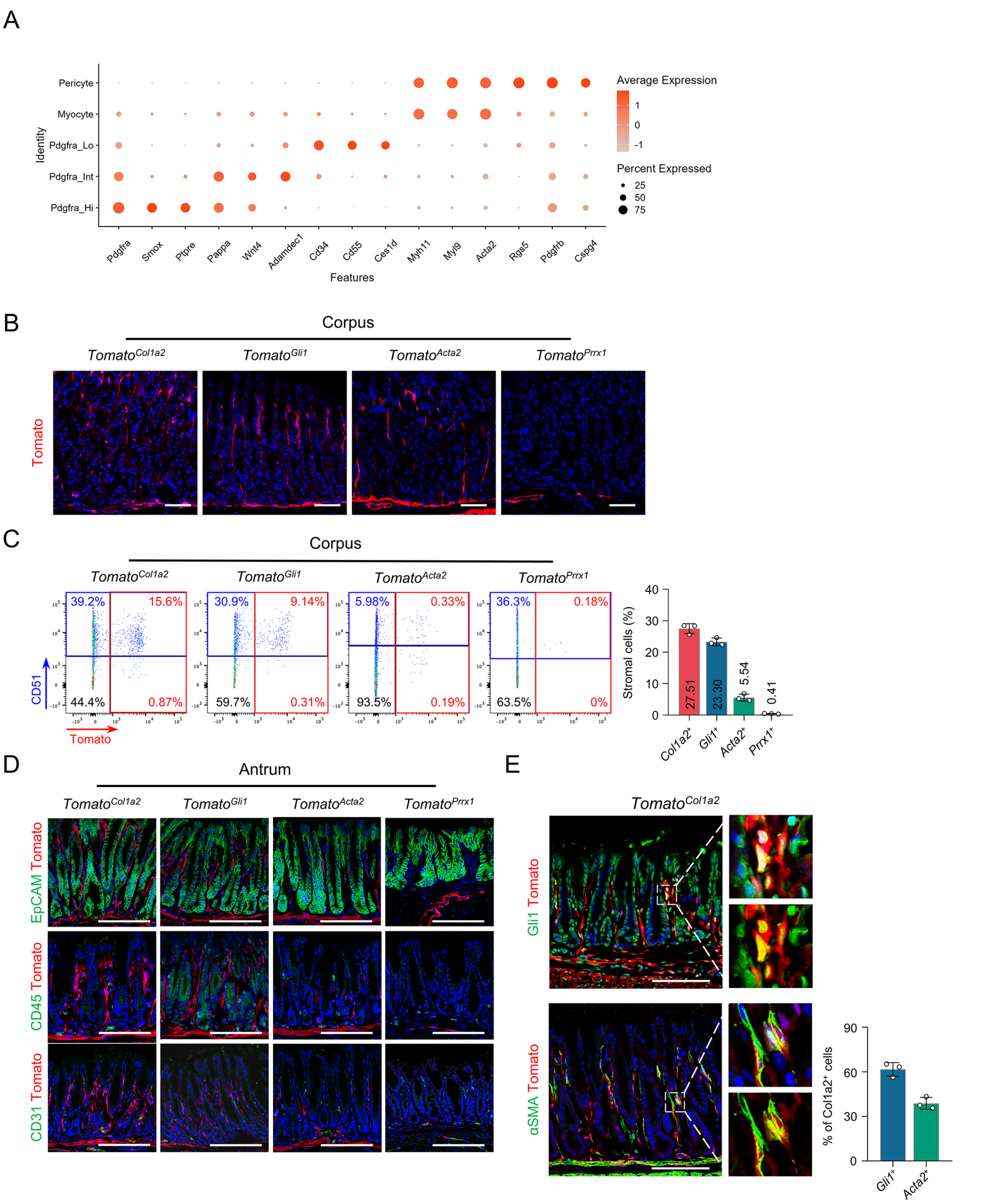

Figure S2. Genetic tracing analyses of stromal cells in mouse antrum and corpus.

A. Markers used to cluster the antral stromal cells.

B. Representative genetic tracing results of the corpus across the four mouse lines (Day 30 post TAM).

C. Flow cytometry analysis of $Tomato^{+}$ in total $CD31^{-}CD45^{-}EpCAM^{-}CD51^{+}$ stromal cells in the corpus of the four mouse lines (Day 30 post TAM). Right panels: quantitation results. n=3 mice.

D. Immunostaining for CD31, EpCAM, or CD45, on antrum sections of the four mouse lines (30 days post TAM).

E. Representative immunostaining results for Gli1 or αSMA on antrum sections of *Bmpr1a*^{Col1a2} mice. Right panels: Quantitation results. n=3 mice.

Scale bars: 100 μm.

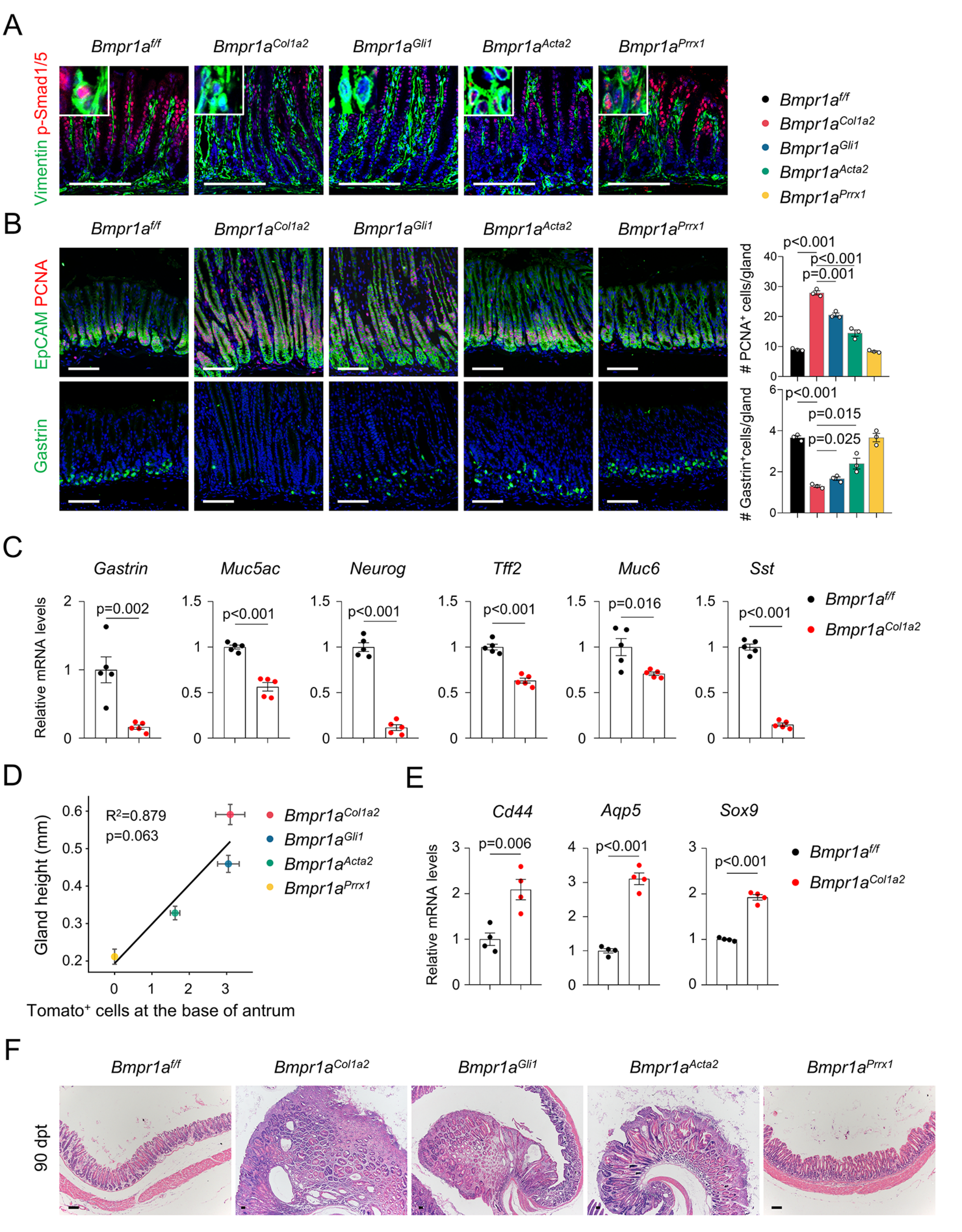

Figure S3. Stromal *Bmpr1a* ablation promotes proliferation but inhibits differentiation of GSCs.

A. Representative immunostaining results of p-Smad1/5 on antrum sections of the four *Bmpr1a* ablation mouse lines (Day 14 post TAM).

B. Representative H/E and immunostaining results of gastrin and EpCAM/PNCA on antrum sections of $Bmpr1a^{Col1a2}$ mice. Right panels: quantitative data of the number of $PCNA^+$ cells and $gastrin^+$ cells per gland, n=3 mice.

C. qPCR analysis of the differentiation marker expression in antrum samples of *Bmpr1a*^{Col1a2} mice. n=5 mice.

D. Correlations between gland height and the stromal population size at the base. n=3 mice for each genotype.

E. qPCR analysis of GSC marker expression in antrum samples of *Bmpr1a*^{Col1a2} mice, with control values set at 1.0. n=4 mice.

F. Representative H/E staining results of antrum sections on Day 90 after TAM administration.

Unpaired two-tailed Student's t test was applied for (B), (C) and (E). Linear regression analysis was applied for (D). $p<0.05$ was considered statistically significant. Scale bars: 100 μm.

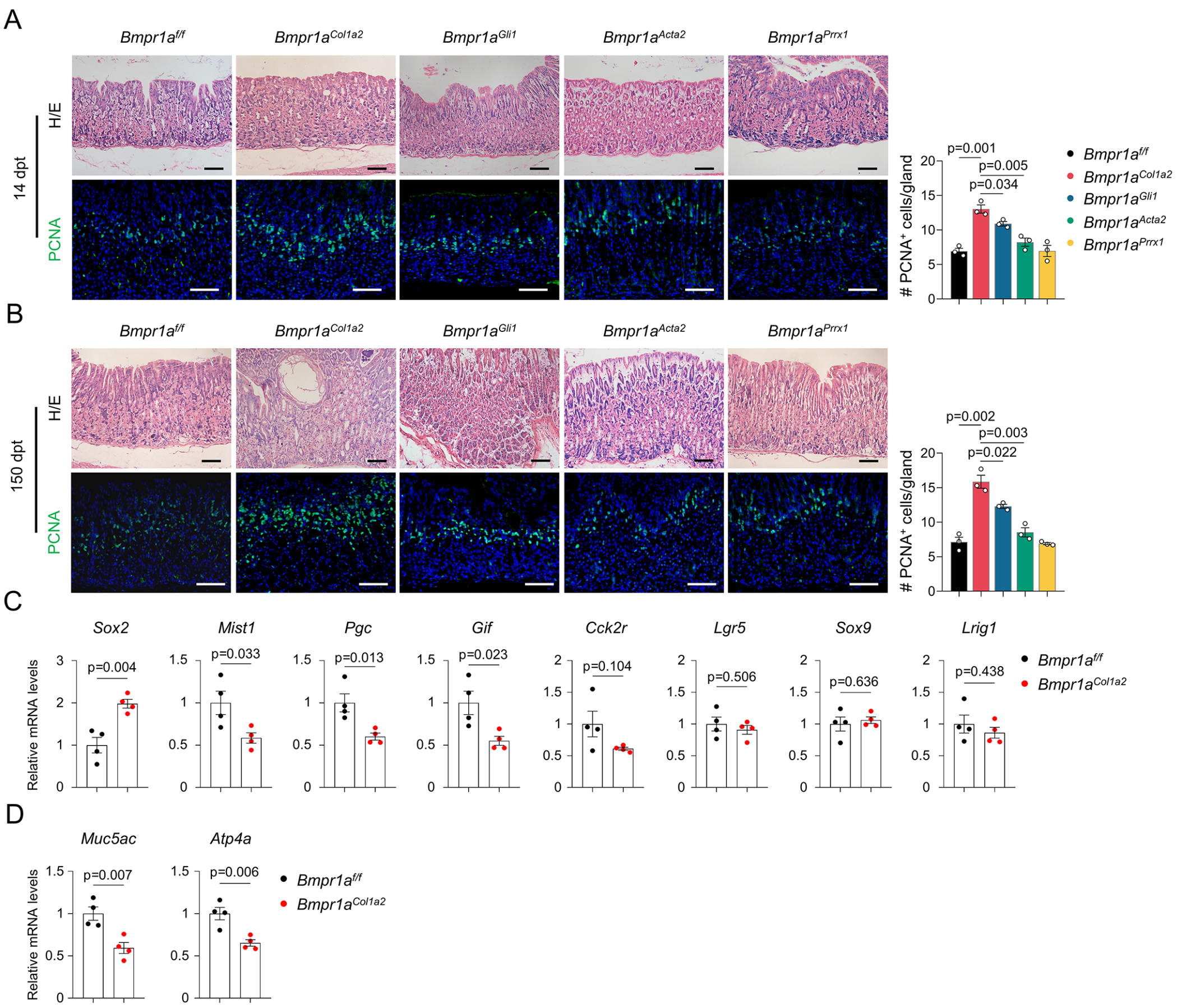

Figure S4. The corpus phenotypes of *Bmpr1a* ablation lines.

A. Representative H/E staining and PCNA immunostaining results of the corpus in mice at day 14 post TAM. Right panels: quantitation data of $PCNA^+$ cells/gland. n=3 mice.

B. Representative H/E staining and PCNA immunostaining results of the corpus in mice 5 months post TAM. Right panels: quantitation data of $PCNA^+$ cells/gland. n=3 mice.

C. qPCR analysis of the GSC marker expression in the corpus. n=4 mice.

D. qPCR analysis of differentiation marker expression in the corpus. n=4 mice.

Unpaired two-tailed Student's t test was applied for (A-D). $p<0.05$ was considered statistically significant. Scale bars: 100 μm.

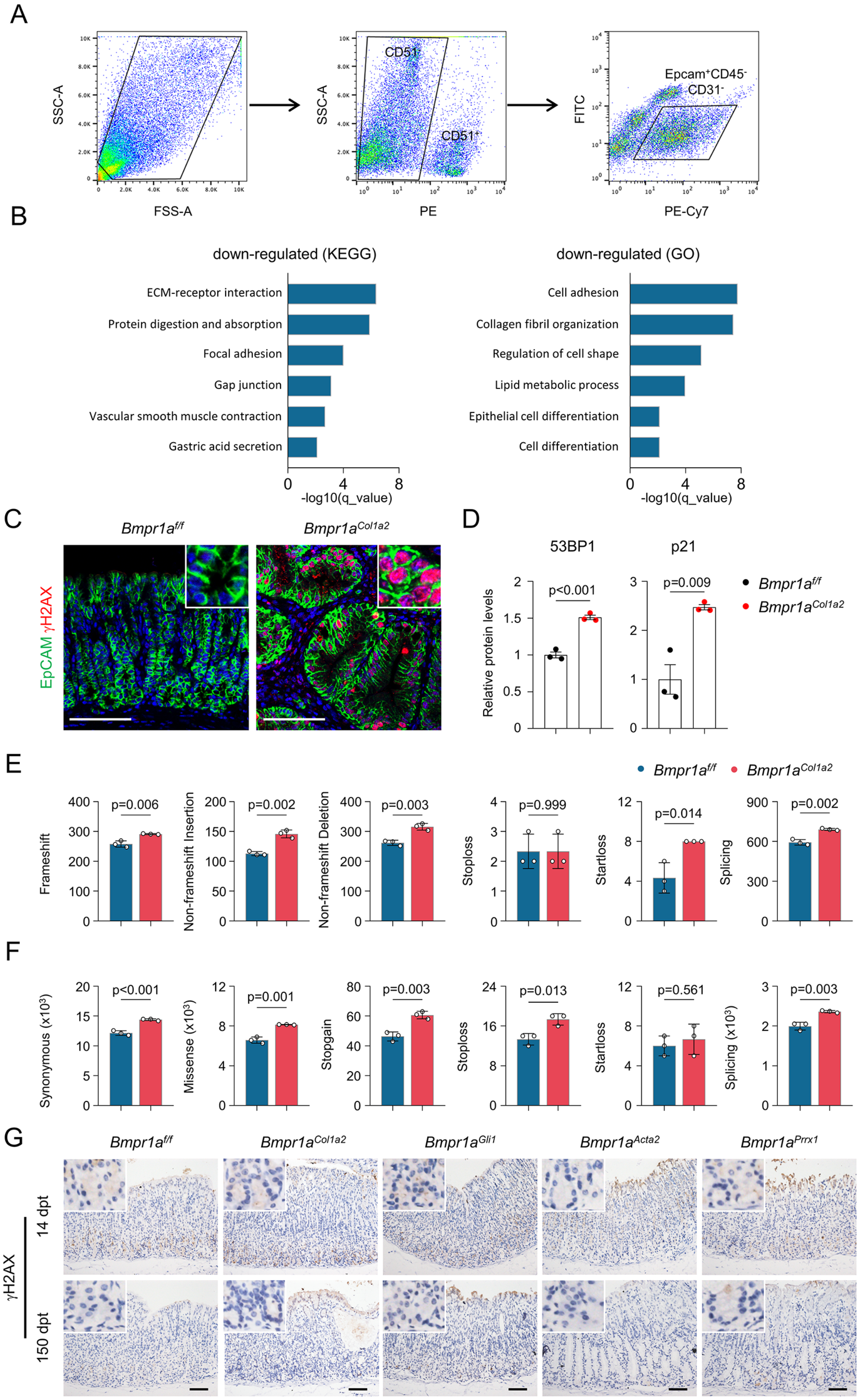

Figure S5. Analysis of the transcriptome and DNA damage in gastric epithelial cells.

A. The gating strategies for FACS sorting of antral epithelial cells.

B. Bulk RNA-seq results of down-regulated genes in antral epithelial cells of *Bmpr1a*^{Col1a2} mice.

C. Representative images of co-immunostaining for γH2AX and EpCAM on antrum sections of *Bmpr1a*^{Col1a2} mice.

D. Quantitation data for 53BP1 and p21 (Figure 5E). n=3 mice.

E. The numbers of Indels in *Bmpr1a*^{Col1a2} and control mice.

F. The numbers of point mutations in *Bmpr1a*^{Col1a2} and control mice.

G. Representative γH2AX staining results of corpus sections of the four *Bmpr1a* ablation mice at day 14 or 150 post TAM.

Unpaired two-tailed Student's t test was applied for (D), (E) and (F). $p<0.05$ was considered statistically significant. Scale bars: 100 μm.

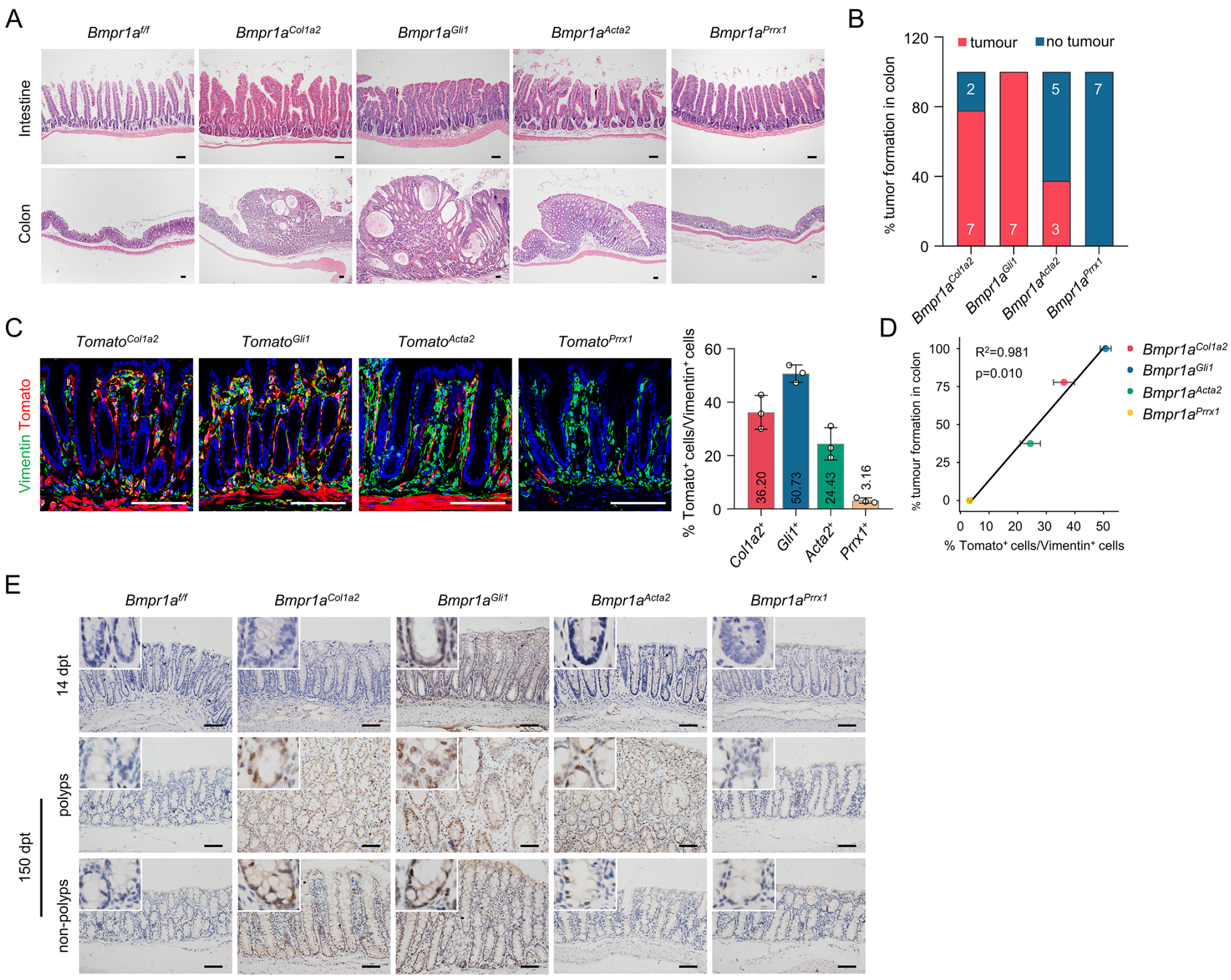

Figure S6. Stromal *Bmpr1a* ablation induces DNA damage in ISCs.

A. H/E staining results of the small intestine and colon at day 150 post TAM.

B. Percentages of mouse lines that developed polyps at day 150 post TAM, with numbers indicating the numbers of mice.

C. Tracing results of the 4 lineage cells in the colon of adult mice (Day 30 post TAM). The colon sections were immunostaining for Vimentin. Right panels: quantitation data for the percentages of Tomato$^+$ cells in Vimentin$^+$ stromal cells. n=3 mice.

D. Correlation between stromal population size and polyp incidence in the colon across the four *Bmpr1a* ablation models. n=3 mice for each genotype.

E. Representative H2AX staining results of colon sections of the four *Bmpr1a* ablation mouse lines at day 14 or 150 post TAM.

Linear regression analysis was applied for (D). Scale bars: 100 μm.

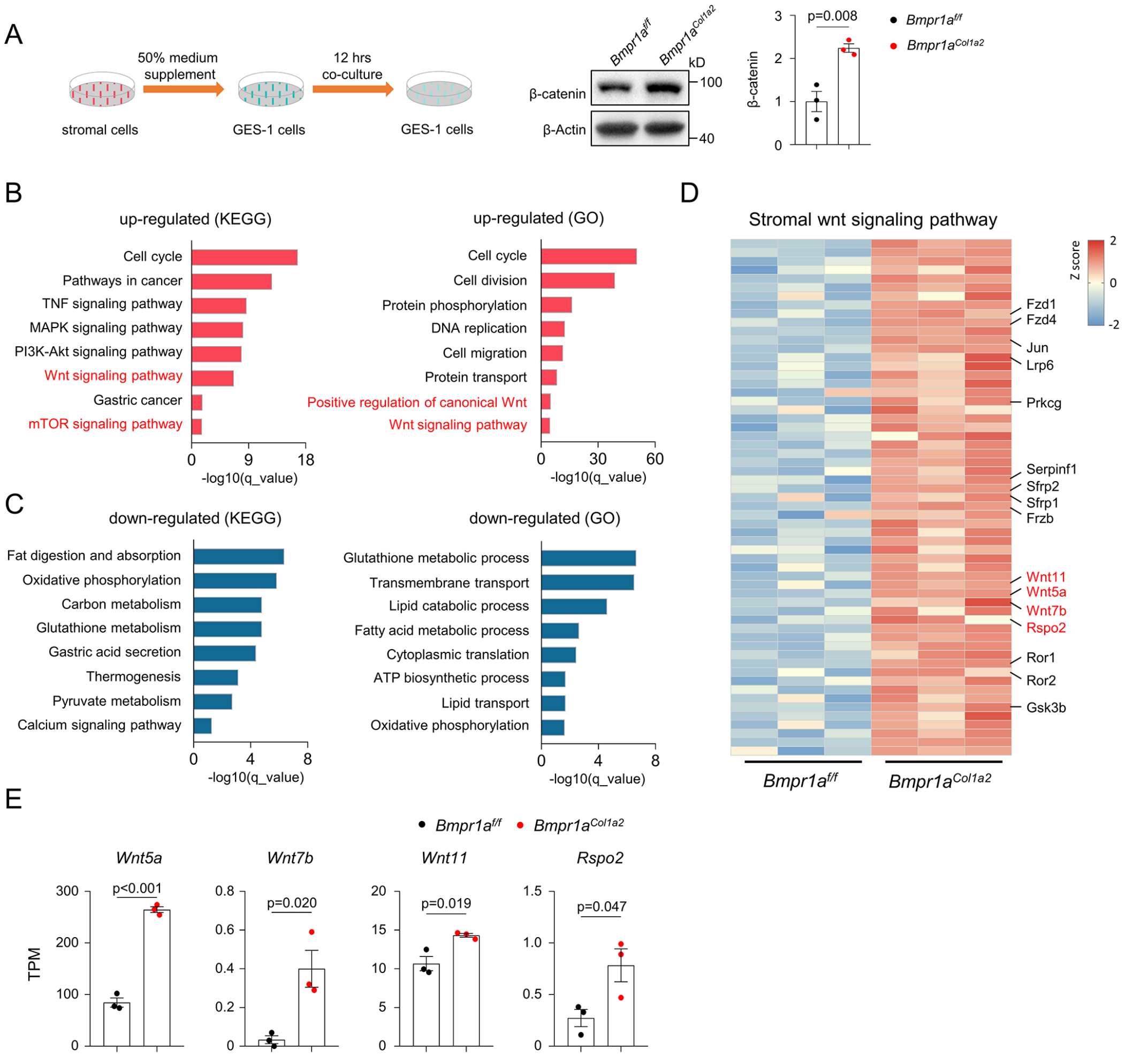

Figure S7. Analysis of Wnt/Rspo expression in *Bmpr1a*$^{-/-}$ stromal cells.

A. Culture of GES-1 with 50% medium of stromal cells derived from *Bmpr1a*^{Col1a2} mice led to elevated β-catenin compared to stromal cells from control mice. Right panels: quantitation data. n=3.

B. KEGG and GO analysis results showing the up-regulated pathways or modules in *Bmpr1a*$^{-/-}$ stromal cells compared to control cells.

C. KEGG and GO analysis results showing the down-regulated pathways or modules in *Bmpr1a*$^{-/-}$ stromal cells compared to control cells.

D. A heatmap illustrates the enrichment of Wnt signalling pathway genes in *Bmpr1a*$^{-/-}$ stromal cells.

E. TPM values for *Wnt5a*, *7b*, and *11* and *Rspo2*. n=3 mice.

Unpaired two-tailed Student's t test was applied for (A) and (E). p<0.05 was considered statistically significant.

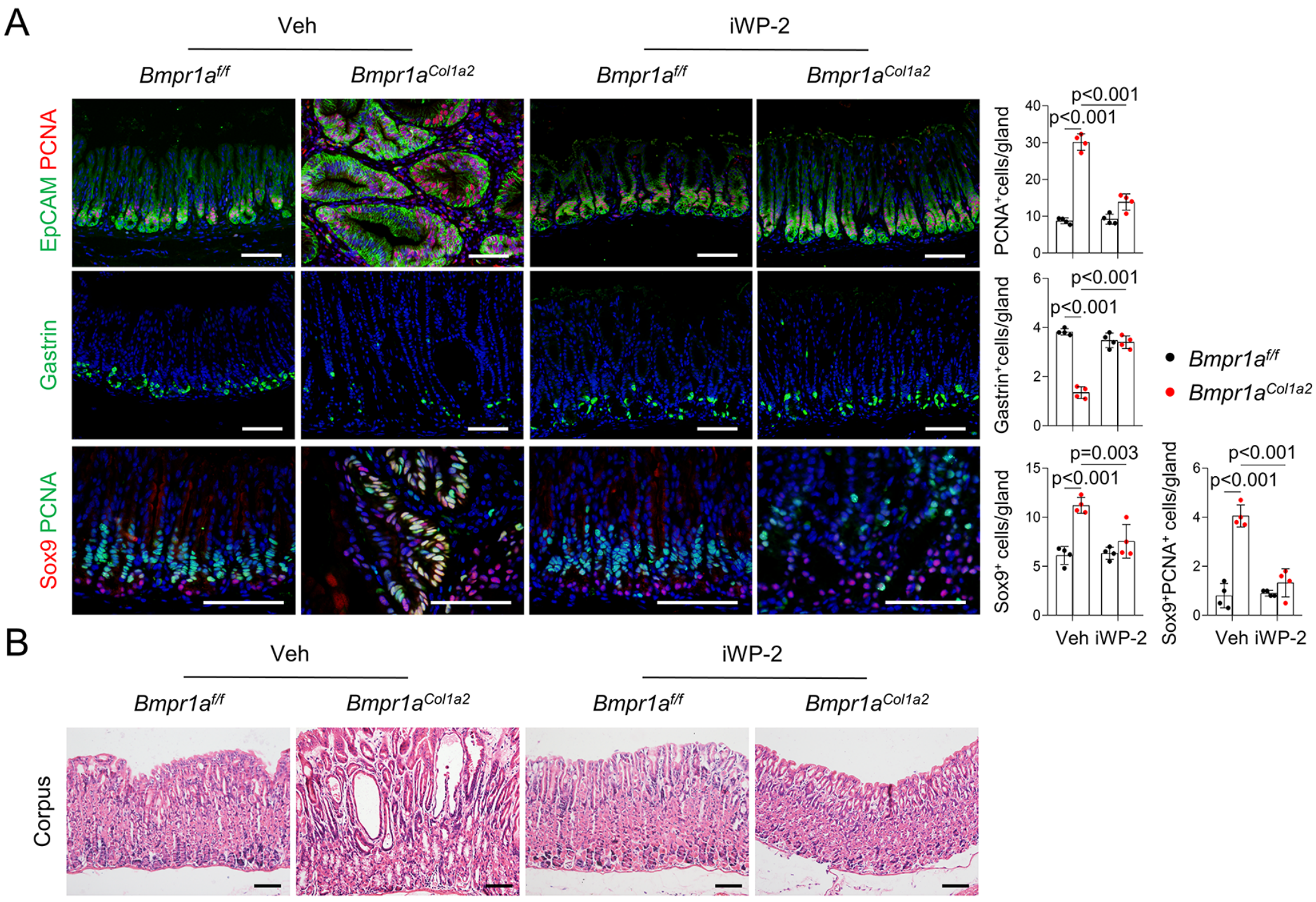

Figure S8. Wnt secretion is required for antral and corpus hyperplasia.

A. Representative EpCAM/PCNA, gastrin, or Sox9/PNCA immunostaining results showing the effect of iWP-2 on *Bmpr1a^{Col1a2}* mice at day 150 post TAM. Right panels: quantitation data. n=4 mice.

B. Representative H/E staining results showing that iWP-2 prevented epithelial overproliferation in the corpus of *Bmpr1a^{Col1a2}* mice.

Two-way ANOVA was applied for (A). $p<0.05$ was considered statistically significant. Scale bars: 100 μm.

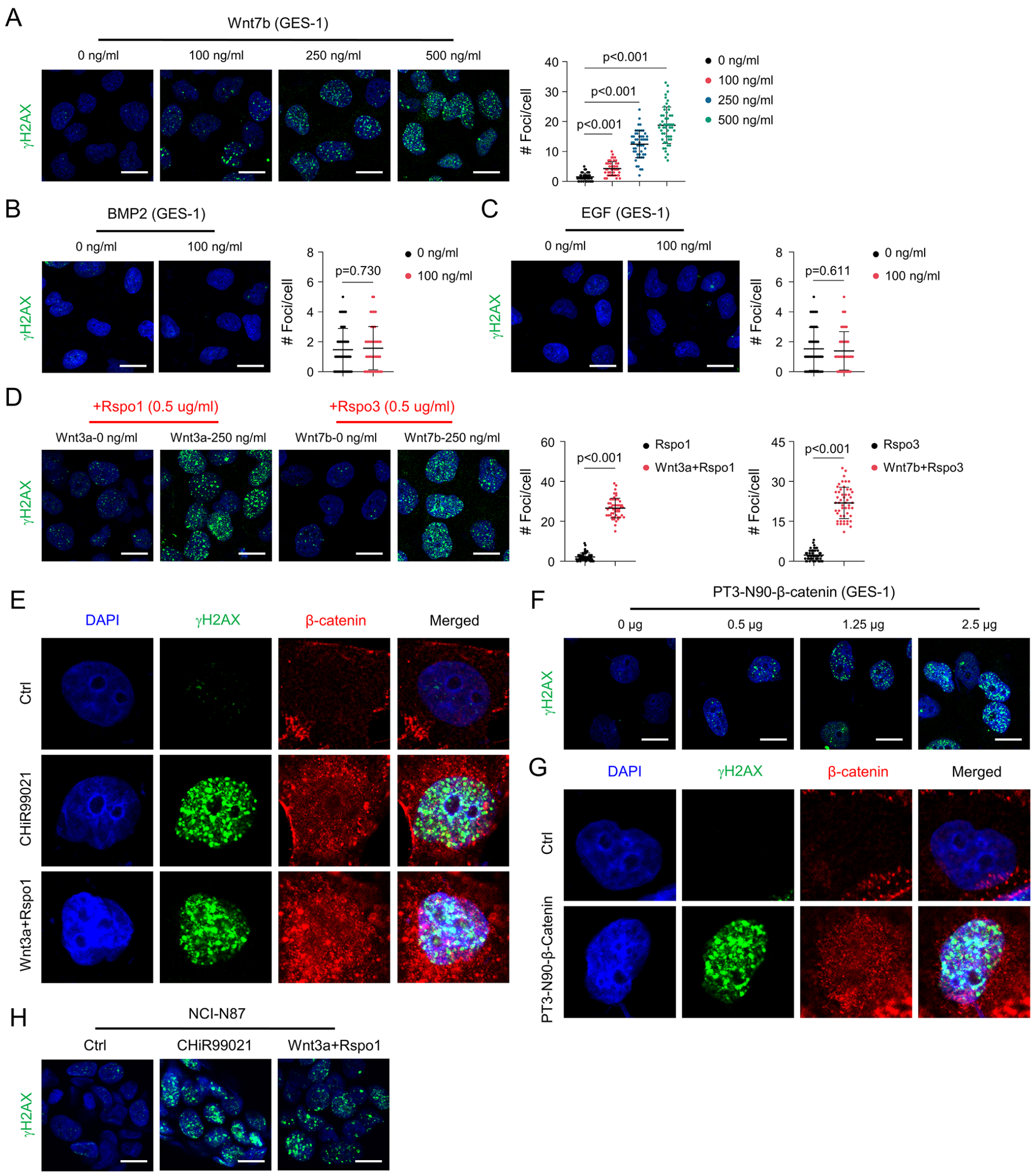

Figure S9. Elevated Wnt/β-catenin induces DNA damage.

A. Wnt7b induced DNA foci formation in GES-1 cells in a dose-dependent manner after 12 hrs of treatment. Right panels: quantification data from 3 repeated experiments.

B. BMP2 had no effect on DNA foci formation in GES-1 cells. Right panels: quantification data from 3 repeated experiments.

C. EGF had no effect on DNA foci formation in GES-1 cells. Right panels: quantification data from 3 repeated experiments.

D. Wnt3a combine with Rspo1, or Wnt7b combined with Rspo3, exhibited synergistic effect on DNA damage foci formation in GES-1 cells. Refer to Figure 7B for Wnt3a

treatment results and Figure S9A for Wnt7b treatment. Right panels: quantification data from 3 repeated experiments.

E. β-catenin did not co-localize with DNA damage foci in response to Wnt activation in GES-1 cells.

F. Ectopically expressed β-catenin induced foci formation in GES-1 cells transfected with PT3-N90-β-catenin plasmid for 24 hrs.

G. Ectopically expressed β-catenin did not co-localize with DNA damage foci in GES-1 cells.

H. NCI-N87 cells showed DNA damage foci formation in response to Wnt/β-catenin activation.

Unpaired two-tailed Student's t test was applied for (A-D). $p<0.05$ was considered statistically significant. Scale bars: 300 μm.

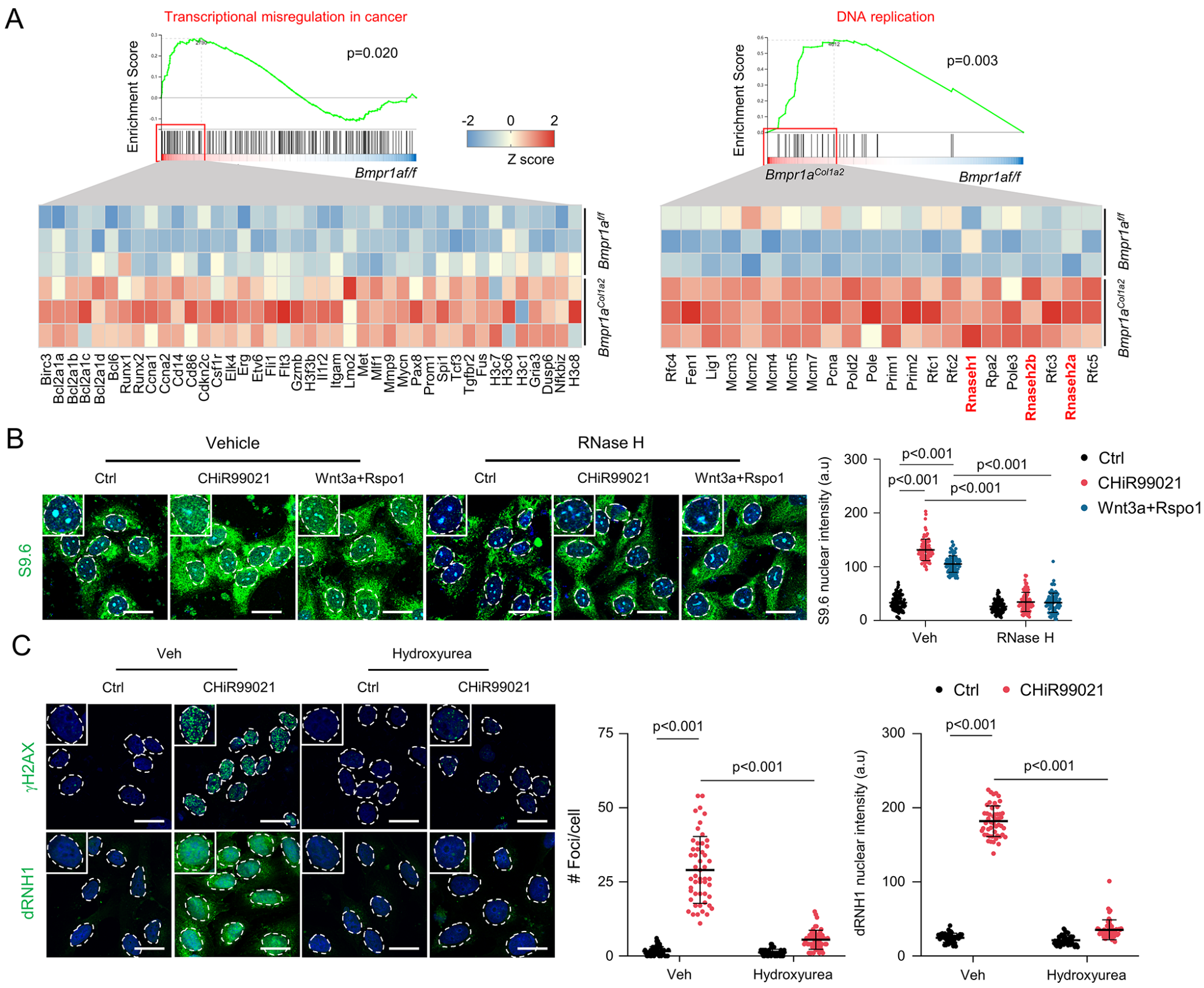

Figure S10. Elevated Wnt/β-catenin induces R-loop formation.

A. GSEA analysis revealed DNA replication and transcriptional misregulation in cancer in the epithelia of *Bmpr1a*^{Col1a2} mice.

B. GES-1 cells showed R-loop formation in response to Wnt activation, as judged by S9.6 immunostaining, which was abolished by RNase H treatment; cytosolic signals were deemed non-specific. Right panels: quantification data of nuclear S9.6 intensity from 3 repeated experiments.

C. Low dose of hydroxyurea (HU, 0.01 mM) inhibited both DNA damage foci formation and R-loop formation in GES-1 cells. The cells were treated first with HU for 12 hrs and then with CHiR99021 for another 12 hrs. Right panels: quantification data from 3 repeated experiments.

Unpaired two-tailed Student's t test was applied for (B) and (C). $p<0.05$ was considered statistically significant. Scale bars: 300 μm.

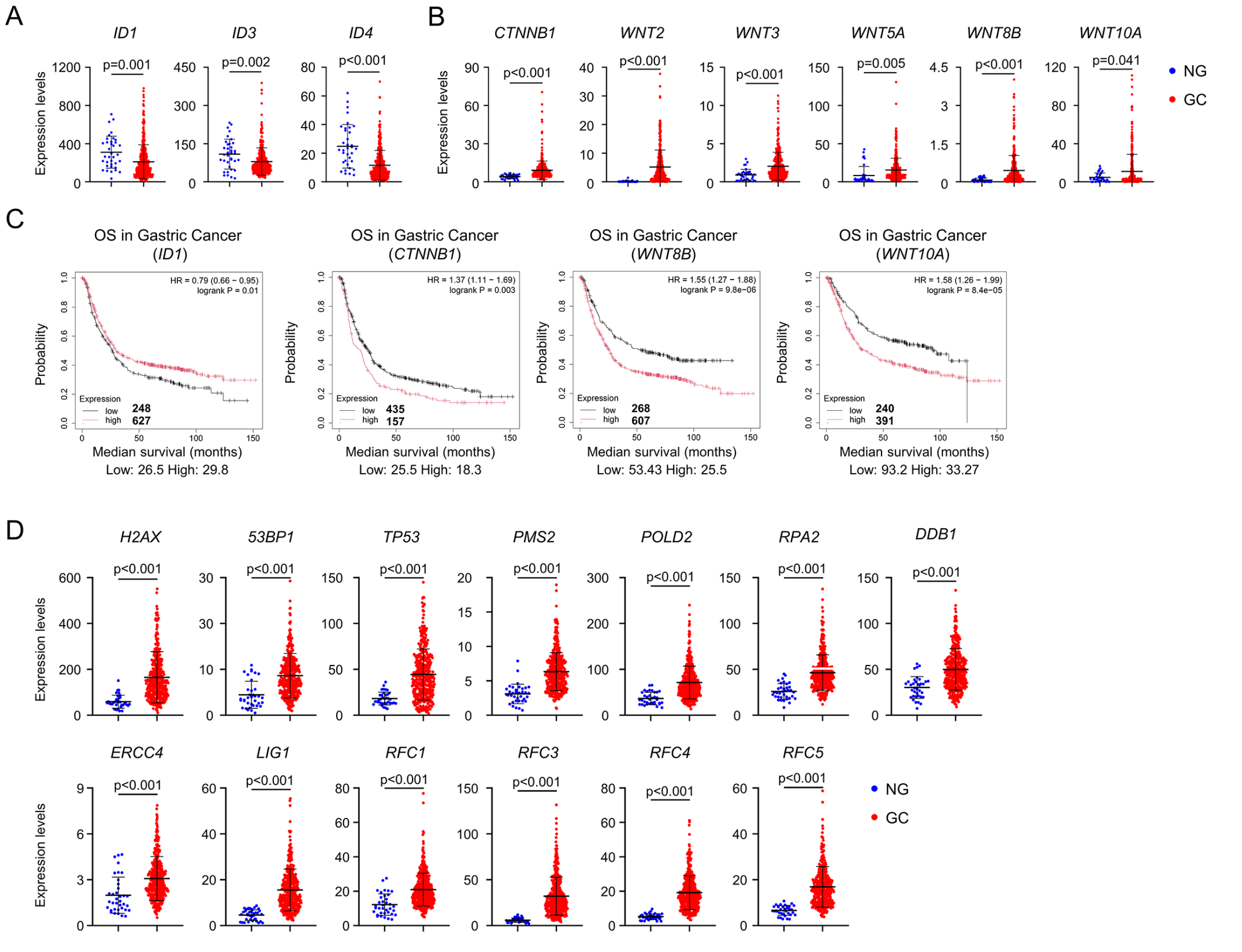

Figure S11. Expression of BMP and Wnt pathway and DNA repair genes in human GC samples.

A. Analysis of the TCGA data revealed elevated expression of BMP pathway genes in GC patient samples. NG=36, GC=410.

B. Analysis of the TCGA data revealed elevated expression of Wnt pathway genes in GC patient samples. NG=36, GC=410.

C. The correlation between GC patient prognosis and the expression levels of BMP or Wnt signalling molecules.

D. The DNA repair genes induced in antral epithelial cells of *Bmpr1a*^{Col1a2} mice were expressed at higher levels in patient samples compared to control samples. NG=36, GC=410.

Unpaired two-tailed Student's t test was applied for (A), (B) and (D). $p<0.05$ was considered statistically significant. Scale bars: 100 µm.

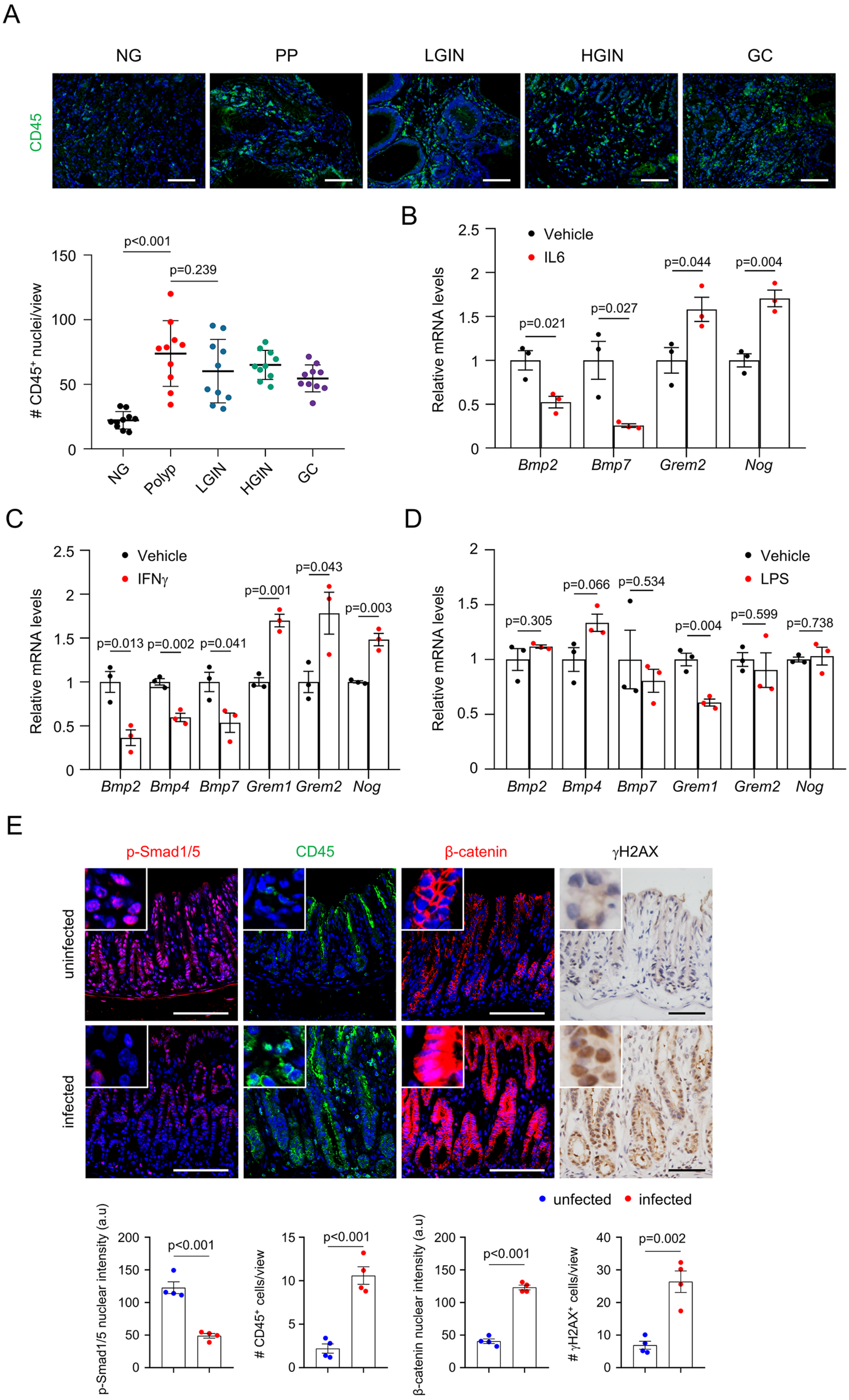
A
NG
PP
LGIN
HGIN
GC
CD45
CD45⁺ nuclei/view
p<0.001
p=0.239
Polyp
B
Relative mRNA levels
Vehicle
IL6
p=0.021
p=0.027
p=0.044
p=0.004
Bmp2
Bmp7
Grem2
Nog
C
Vehicle
IFNγ
p=0.013
p=0.002
p=0.041
p=0.001
p=0.043
p=0.003
Bmp2
Bmp4
Bmp7
Grem1
Grem2
Nog
D
Vehicle
LPS
p=0.305
p=0.066
p=0.534
p=0.004
p=0.599
p=0.738
E
p-Smad1/5
CD45
β-catenin
γH2AX
uninfected
infected
unfected
infected
p-Smad1/5 nuclear intensity (a.u)
CD45⁺ cells/view
β-catenin nuclear intensity (a.u)
γH2AX⁺ cells/view
p<0.001
p<0.001
p<0.001
p=0.002

Figure S12. The effects of inflammation on the expression of BMP ligands and antagonists in gastric stromal or epithelial cells.

A. Immunostaining for CD45 in patient sample sections. Bottom panels: quantification data. n=10 per stage.

B. qPCR analysis of the levels of BMP ligands and antagonists in gastric stromal cells treated with IL-6, n=3.

C. qPCR analysis of the levels of BMP ligands and antagonists in gastric stromal cells treated with IFNγ. n=3.

D. qPCR analysis of the levels of BMP ligands and antagonists in gastric epithelial cells (GES-1) treated with LPS. n=3.

E. Representative immunostaining results for p-Smad1/5, CD45, β-catenin, and γH2AX in *H. pylori*-induced chronic gastritis mouse model. Bottom panels: Quantification data. n=4.

Unpaired two-tailed Student's t test was applied for (A-E). $p<0.05$ was considered statistically significant.
