# Supplementary material for: Stromal BMP signalling maintains stem cell genome integrity and prevents gastric cancer initiation
Source: Cell Death Dis. 2026 Jul 17;17(1):882. doi: 10.1038/s41419-026-09088-z (PMC13658785; doi:10.1038/s41419-026-09088-z)

Fig. 5E

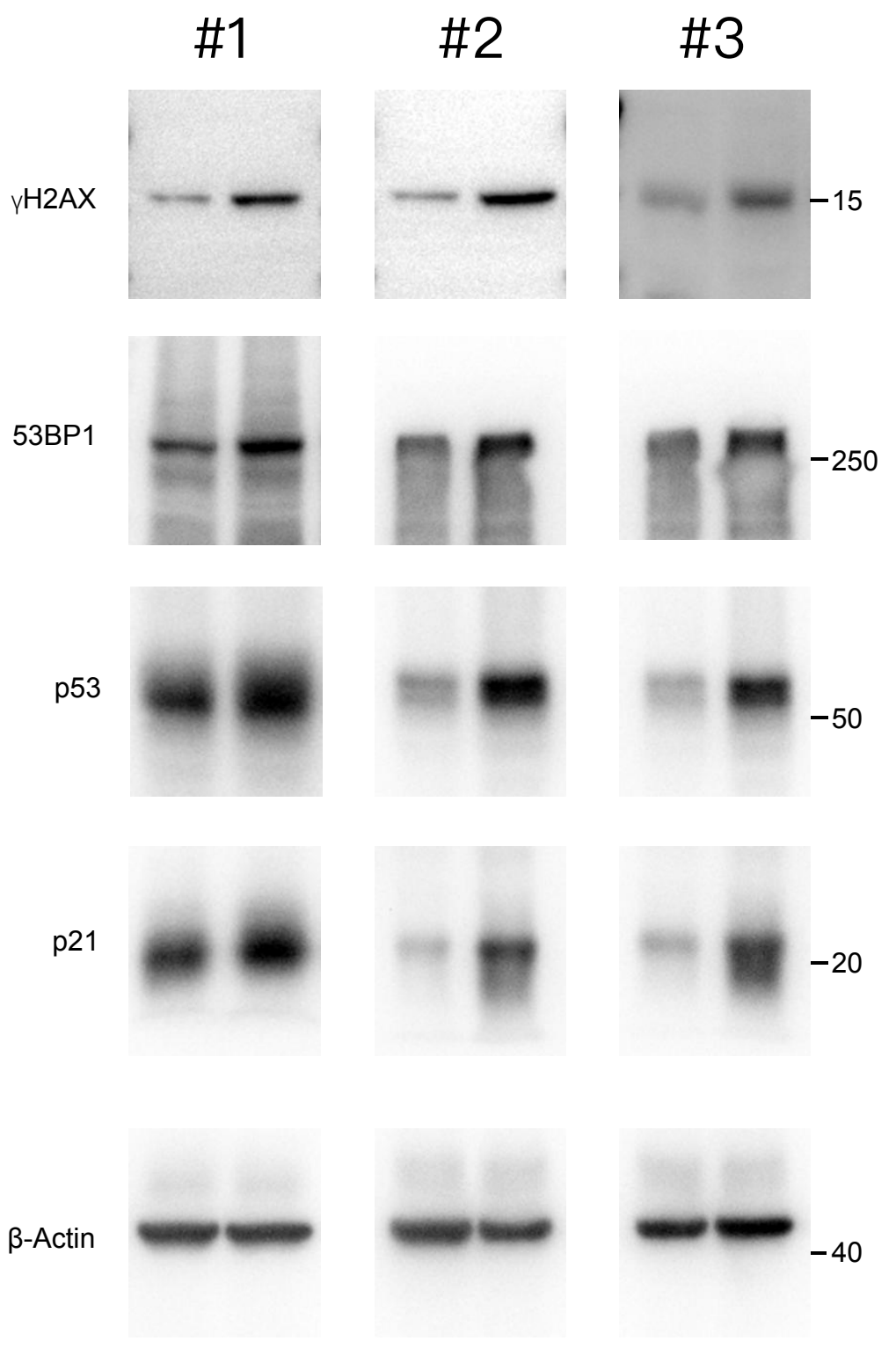

Fig. 5F

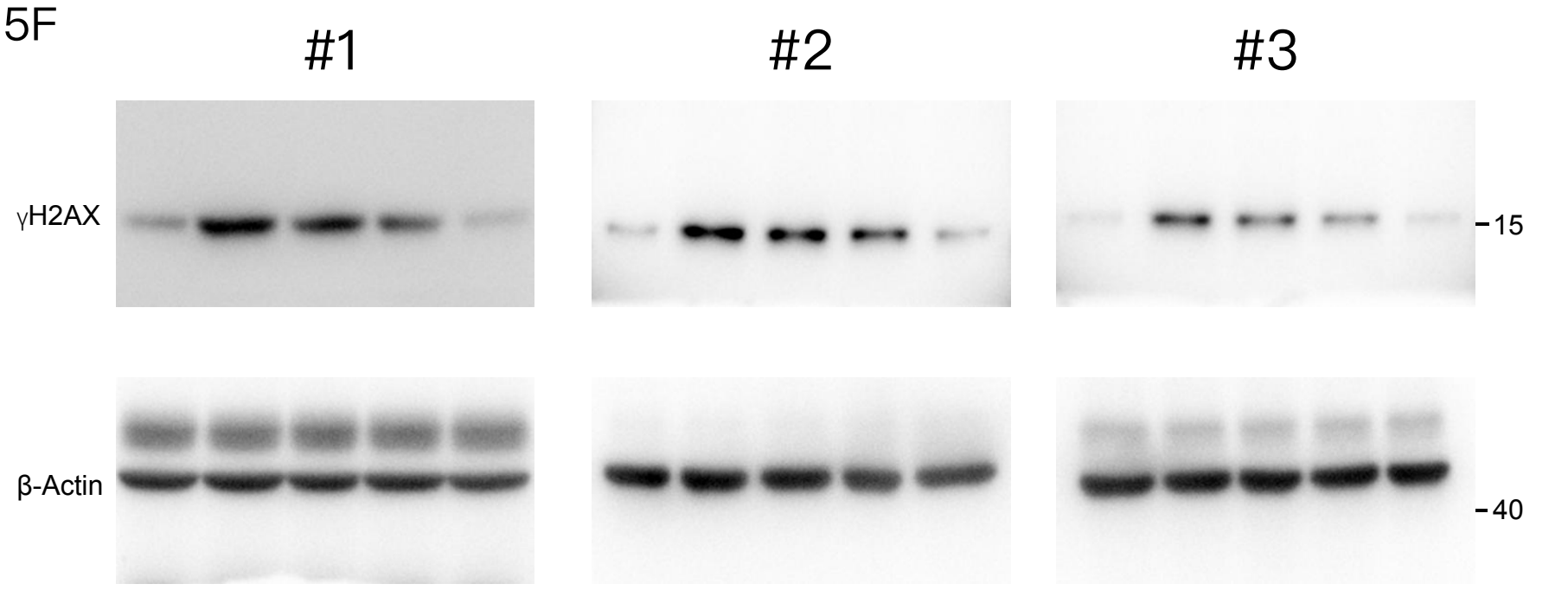

Fig. 6C

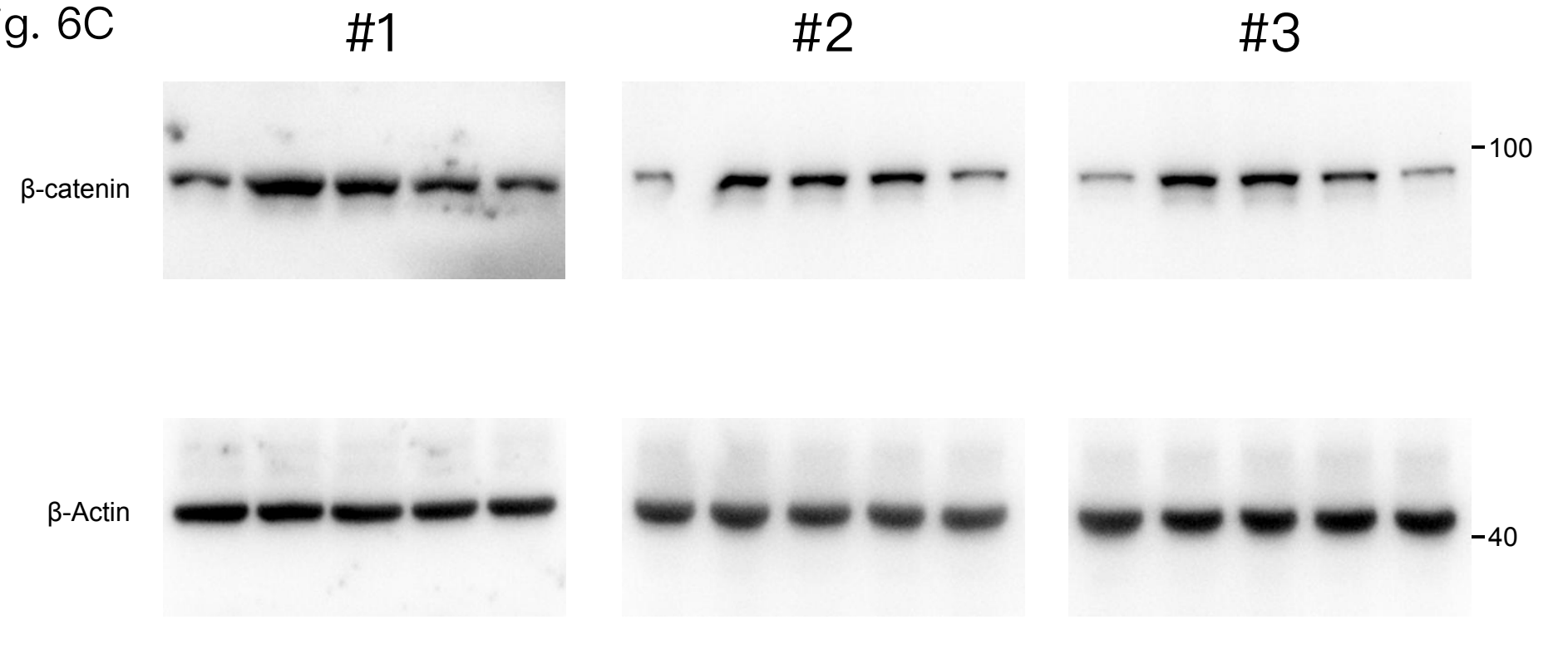

Fig. 6E

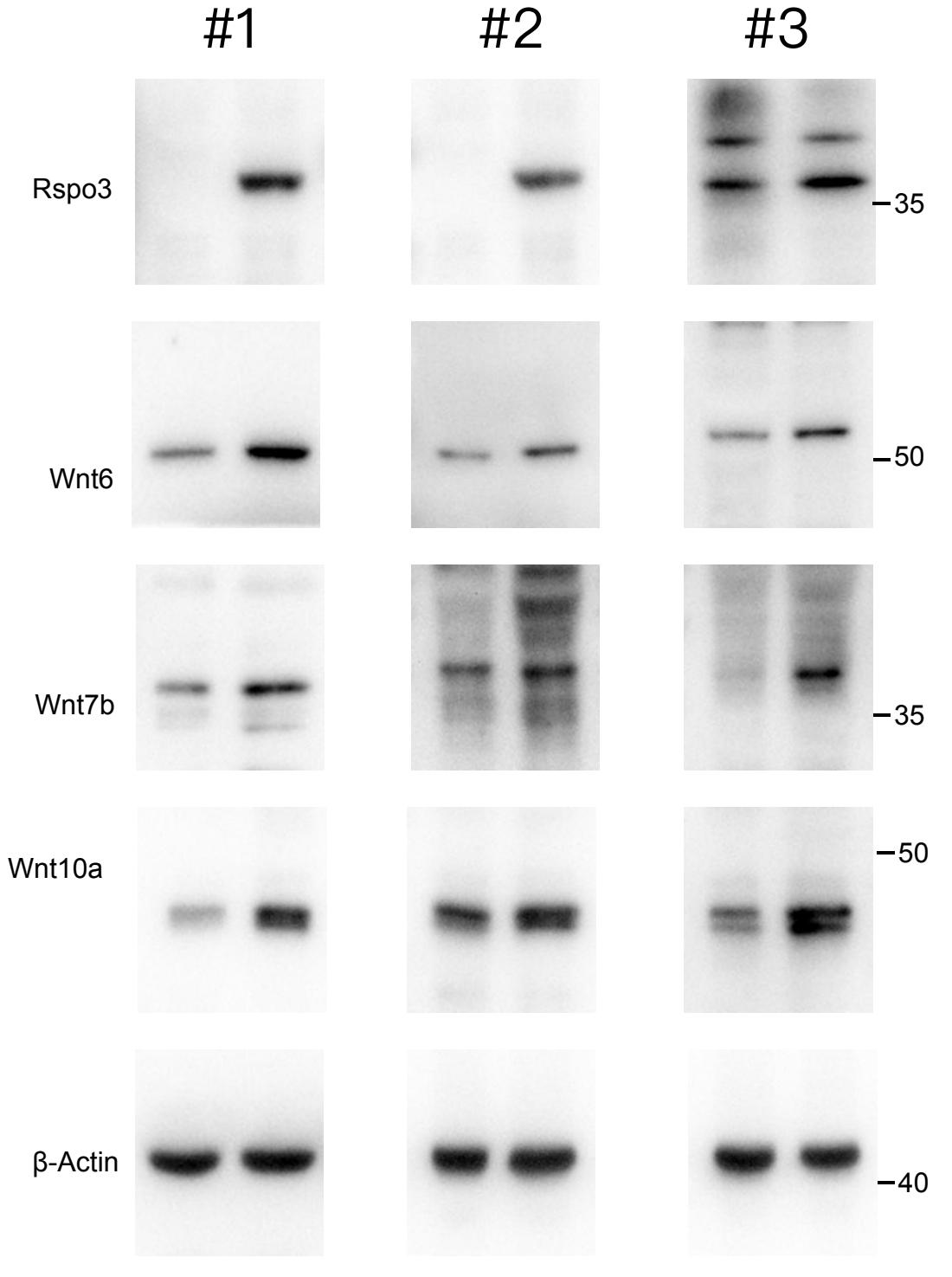

#1
#2
#3
Rspo3
Wnt6
Wnt7b
Wnt10a
β-Actin
35
50
35
50
40

Fig. 6G

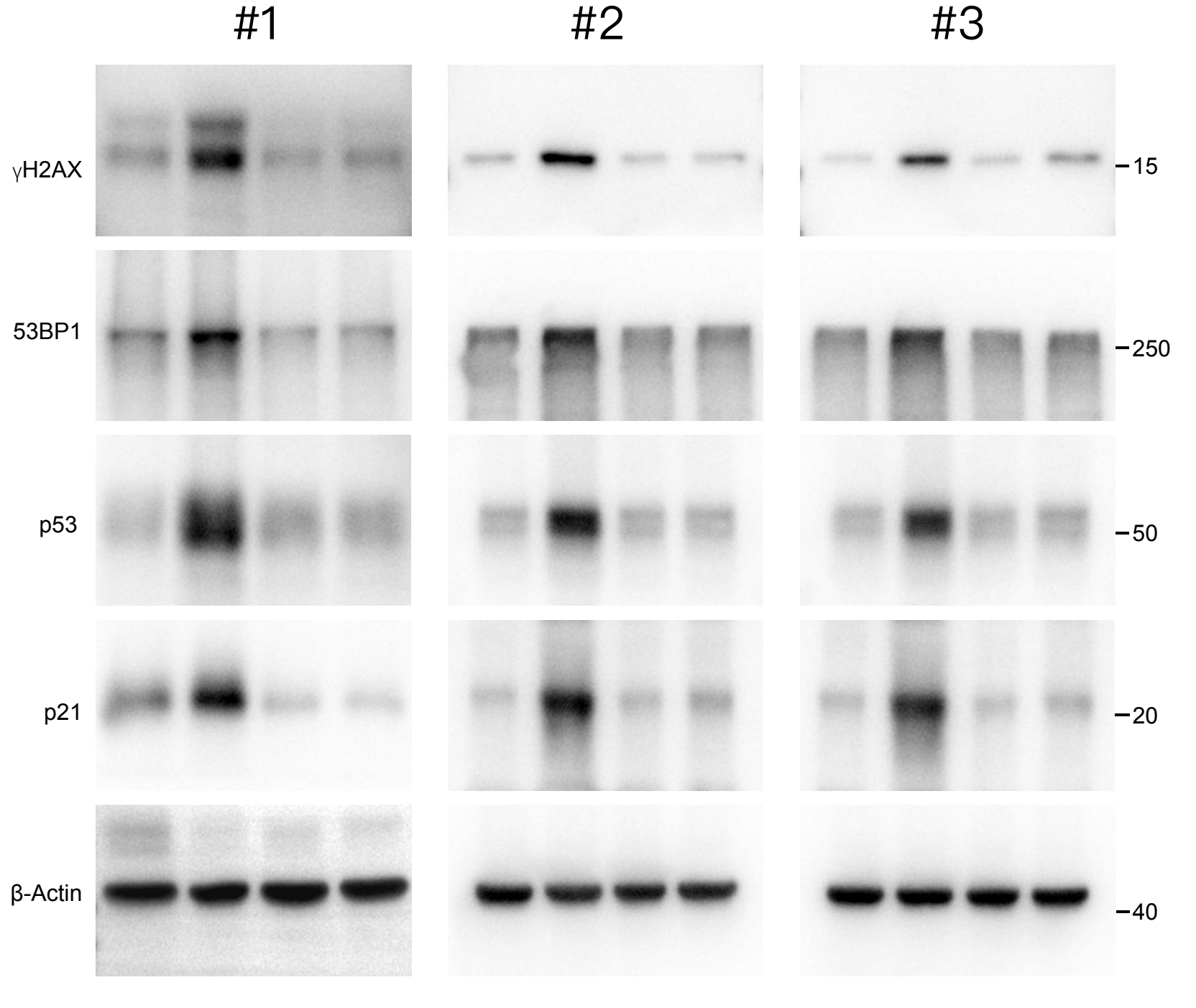

#1
#2
#3
γH2AX
53BP1
p53
p21
β-Actin
15
250
50
20
40

Fig. S7A

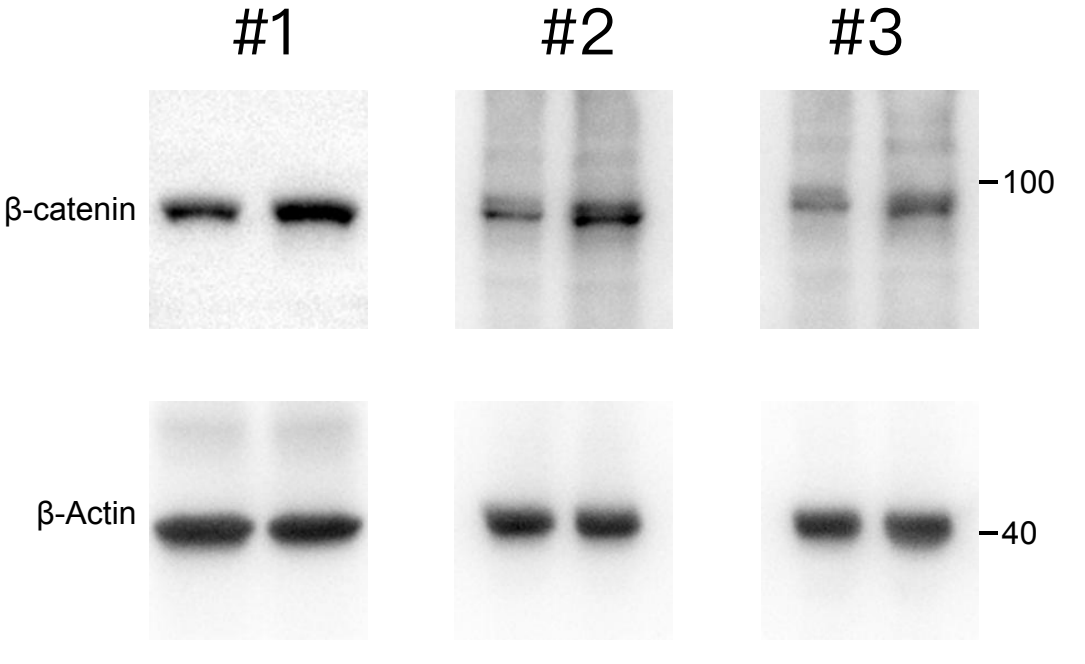

Supplement: Supplementary file 2 [file 41419_2026_9088_MOESM2_ESM.pdf]
